# Electronically Coupled, Cofacially Linked, Hypervalent Antimony(V) Porphyrin Homodimer: Synthesis, Spectroscopy, and Photochemistry

**DOI:** 10.1021/acs.inorgchem.5c05959

**Published:** 2026-03-24

**Authors:** Prashanth K. Poddutoori, Peyton Ellis, Jatan K. Sharma, Niloofar Zarrabi, Art van der Est, Francis D’Souza

**Affiliations:** a Department of Chemistry & Biochemistry, 14713 University of Minnesota Duluth, 1038 University Drive, Duluth, Minnesota 55812, United States; b Department of Chemistry, 3404 University of North Texas, 1155 Union Circle, # 305070, Denton, Texas 76203-5017, United States; c Department of Chemistry, 7497 Brock University, 1812 Sir Isaac Brock Way, St. Catharines, ON L2S 3A1, Canada

## Abstract

A covalently linked cofacial homodimer has been synthesized using hypervalent antimony­(V) porphyrins. The two porphyrins are connected by an −OCH_2_O– bridge, maintaining a distance of 5.85 Å between the Sb centers. Despite the positive charge on the porphyrin entities, the resulting homodimer is structurally stable, with minimal or no repulsive forces between them. Density functional theory (DFT)-optimized structures show that the two porphyrins are nearly coplanar with an angle of ∼15° between the two planes. The highest occupied molecular orbital (HOMO) and lowest unoccupied molecular orbital (LUMO) of the ground state of the dimer are delocalized over both porphyrins, indicating the existence of exciton coupling between them. Optical studies support this observation, suggesting H-type exciton coupling based on the spectral shift of the Soret band. Similar behavior is observed in electrochemical studies as well. Results from the femtosecond transient absorption studies support the existence of exciton coupling, while the triplet-state lifetimes measured by nanosecond transient absorption studies show only minor differences. Time-resolved electron paramagnetic resonance and DFT studies of the lowest triplet state show that it is localized on a single porphyrin. While the localization of the triplet state is consistent with the expected weak coupling between the triplet states due to their small transition dipoles, the optical data, DFT calculations, and structure of the dimer all indicate that the dipole–dipole model of the excitonic coupling is not capable of adequately describing the excited-state properties. Notably, the role of p-block Sb in cofacial porphyrin dimer promoting exciton coupling, irrespective of its higher oxidation state of +5, is borne out from this study.

## Graphical abstract

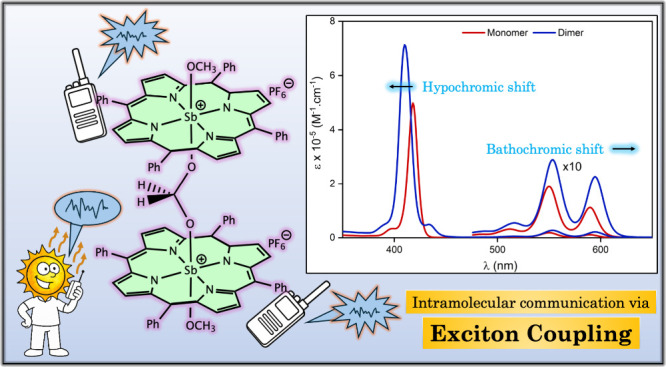

## Introduction

The conversion of sunlight to chemical energy during photosynthesis relies on a series of light-dependent reactions in specialized protein complexes known as reaction centers.^1–6^ This process involves many biochemical components, each serving a unique purpose. Their coordinated actions accomplish the oxidation of a variety of compounds, such as water and reduced sulfur compounds, and the reduction of quinones and ferredoxins, thus converting sunlight energy into chemical energy. Among these components, the chlorophyll dimer or “special pair” plays an important role, particularly in Photosystem II, where it has an exceptionally high oxidation potential.^5,7,8^ In all reaction centers, light excitation leads to charge separation in which the positive charge is localized on the special pair. Following the initial charge separation, electron transfer through a series of secondary electron acceptors stabilizes the charge separation. The dimer structure of the special pair serves two important functions in this process. First, the cofacial arrangement of the two chlorophyll molecules induces excitonic coupling and a red shift of the Q-band absorption, thereby allowing the special pair to act as an excitation energy trap. Second, this structure results in delocalization of the charge in its oxidized form, which helps stabilize the charge-separated states. Thus, building a synthetic analog to the special pair has been a goal in artificial photosynthesis research for many years.^9–13^ In photosynthetic reaction centers, the two chlorophylls are not bound to one another but are held in a cofacial arrangement by intermolecular interactions and by the surrounding protein. The central issue in creating synthetic analogs of the dimer is maintaining two chlorophyll-like molecules in this arrangement without the aid of the protein, while either fully or partially duplicating the photophysical properties of the special pair.

Porphyrins, which share macrocyclic frameworks with chlorophyll molecules, have been widely used to mimic the primary donor in photosynthetic reaction centers.^14–26^ In most cases, the porphyrin is a monomer, but some cofacial dimeric porphyrin structures have been reported.^27–29^ In many of these complexes, the porphyrins are linked covalently via bridging groups attached to the peripheral β-pyrollic or *meso*-positions.^30,31^ Self-assembled dimers in which the bridging groups are coordinated axially to the central metal of porphyrin have also been reported.^32,33^ Porphyrins containing main-group elements are particularly attractive for constructing such dimers because axial bridging groups can be attached covalently to the central element.^34–42^ However, the optical and electronic properties of such complexes have not been widely studied. Recently, our group reported a dimer consisting of electron-rich octaethylporphyrinatoaluminum­(III) (AlP) and electron-poor octaethyl-porphyrinatophosphorus­(V) (PP), axially linked by a μ-oxo bridge with a center-to-center distance of 3.37 Å.^11^ This system exhibited strong excitonic coupling between the two porphyrins, resulting in charge transfer (CT) absorption. The observed CT character makes the dimer a promising synthetic special pair capable of initiating directional electron transfer. In another example, the same AlP and PP were connected through a phenyl bridge at a distance of 9.50 Å, and this greater separation led to the absence of excitonic coupling between the porphyrin rings.^43^ Optical studies of the excited states showed that both energy and electron transfer occurred between the two porphyrin units. While the properties of both these examples are of interest and may help construct an artificial photosynthetic system, the use of two different porphyrin units means they are not actual mimics of the homodimeric special pair, and the involvement of energy and electron transfer in their photophysics interferes with the study of excitonic coupling between the porphyrins.

To address this limitation, we have selected an hypervalent antimony­(V) porphyrin. The choice of this porphyrin was motivated by its high positive potentials and excellent photophysics. Moreover, it has unique axial-bonding ability, where it can form bonds with two different units in unsymmetrical fashion on both sides of it plane. Recently, a few nonporphyrinic antimony-based chromophores were reported, where their structural and optical properties were exploited toward catalytic reactions.^44–47^ Therefore, these properties make the antimony­(V) porphyrin an ideal candidate for mimicking special pair. With this objective, here we report the synthesis and characterization of a tetraphenylantimony­(V) porphyrin homodimer prepared by a new, straightforward synthesis method that links the porphyrins via an axial methyldioxy bridging group. Linking the porphyrins in this manner holds them in a cofacial arrangement at a distance of 5.85 Å between Sb–Sb centers, which is similar to the Mg–Mg distance (5.7, 6.4, 6.3, and 8.2 Å for P_800_, P_840_, P_700_, and P_680_, respectively)^48,49^ in biological special pairs from different reaction centers. In addition, the photophysics of antimony­(V) porphyrins is well known and is like that of the chlorophyll molecules of the special pair dimer. From UV–visible absorption and fluorescence spectroscopy data, we will demonstrate that the novel synthetic procedure utilized for the construction of the new homodimer yields a system in which the singlet excited states of the dimer are excitonically coupled and delocalized over both porphyrins. In contrast, electron paramagnetic resonance (EPR) data and DFT calculations show that the lowest triplet state is localized on one of the units. This suggests that the electronic coupling between the porphyrin units involves the interaction of the transition dipoles, which are large for the allowed singlet–singlet excitations but small for the forbidden singlet–triplet excitations. However, and importantly, the band shifts in the absorption spectra and DFT calculations also indicate that delocalization of the frontier orbitals occurs in the singlet state, either through overlap of the porphyrin orbitals or via superexchange via the bridge, revealing the novel photophysical properties.

## Experimental Section

### Synthesis

The chemicals and solvents utilized in this study were purchased from Alfa Aesar, Acros Organics, Sigma-Aldrich, Tokyo Chemical Industry (TCI), or Fisher Chemical and were used as received. Anhydrous solvents were used in all the reactions. Chromatographic materials were purchased from SiliCycle or Sigma-Aldrich. Scheme 1 depicts the synthesis of the homodimer SbP-OCH_2_O-SbP·(PF_6_)_2_. The synthesis of the precursor SbP-OH·PF_6_ and the reference monomers SbP·PF_6_ has been reported previously.^36^ However, for the completeness, the mass (ESI-MS) and NMR (^1^H, ^13^C, ^19^F, ^31^P) data of SbP·PF_6_ are reported in the Supporting Information. During the synthesis or spectroscopic studies, no uncommon hazards are noted.

**Scheme 1:**
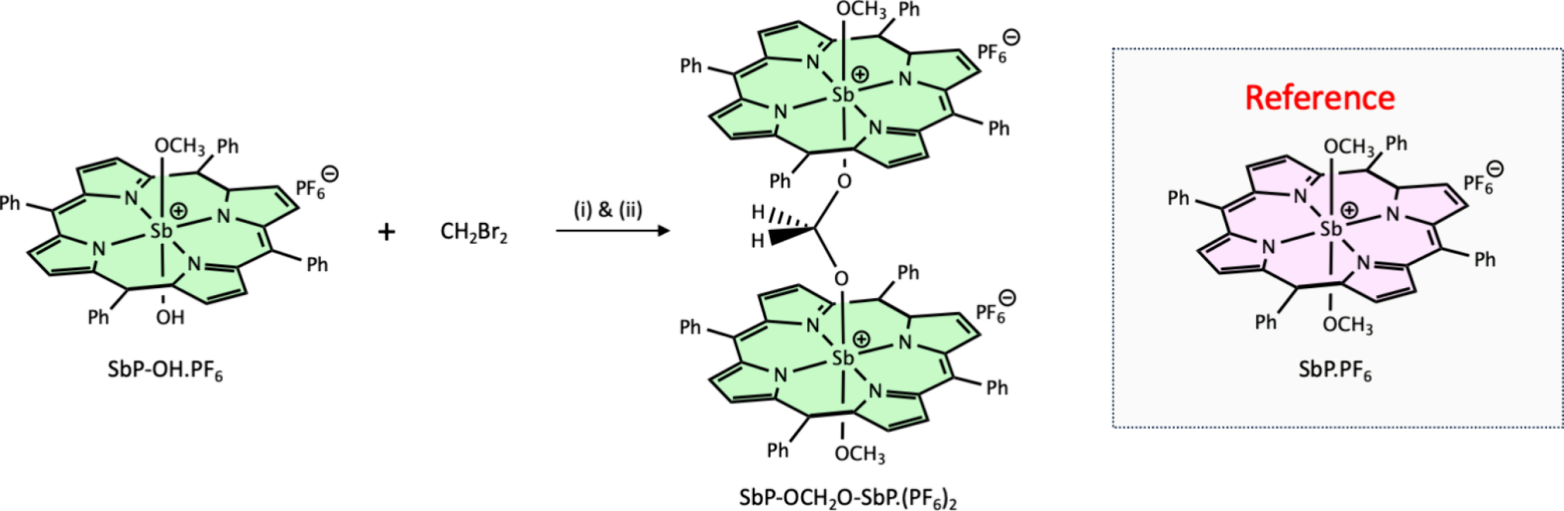
Synthesis of the Homodimer SbP-OCH_2_O-SbP·(PF_6_)_2_ ^a^

a Reaction conditions: (i) dry CH_3_CN, anhydrous K_2_CO_3_, stirring at 60°C under N_2_ for 3.5 h. (ii) NH_4_PF_6._

#### Synthesis of SbP-OCH_2_O-SbP·(PF_6_)_2_

SbP-OH·PF_6_ (29 mg, 0.031 mmol) and K_2_CO_3_ (16 mg, 0.12 mmol) were dissolved in 20 mL of dry CH_3_CN. The reaction mixture was stirred for 5 min while purging with N_2_ at room temperature. At this stage, a large excess of CH_2_Br_2_ (1.0 mL, 14.5 mmol) was added, and the mixture was stirred at 60 °C under a nitrogen atmosphere for 3.5 h. The reaction mixture was then dried and redissolved in CH_3_OH, and NH_4_PF_6_ (100 mg, 0.61 mmol) was added to the suspension to exchange the counterion. The resulting suspension was precipitated by adding water, filtered to collect the precipitate, and air-dried. The crude product was subjected to column chromatography on silica gel. The column was eluted first with CH_2_Cl_2_ to remove the free-base porphyrin and then eluted with CH_2_Cl_2_:EtOAc (94:6–80:20) to obtain the product. The solvent was removed to yield the product in pure form. Yield = 25 mg (86%). ESI-MS: *m*/*z* 788.1560 for [M – 2PF_6_]^2+^, calcd 788.1929 for C_91_H_64_N_8_O_4_Sb_2_^2+^. ^1^H NMR (CDCl_3_, 400 MHz): *δ*, *ppm* 9.15 (s, 16H), 8.00 (bs, 16H), 7.83 (bs, 16H), 7.67 (bs, 8H), −3.10 (s, 6H), −6.19 (s, 2H). ^13^C (CDCl_3_, 100 MHz): *δ*, *ppm* 144.63, 137.55, 135.15, 133.40, 129.97, 128.18, 212.83, 44.73. ^31^P NMR (CDCl_3_, 162 MHz): *δ*, *ppm* −144.68 (sept, 1P, *J* = 713 Hz). ^19^F (CDCl_3,_ 376 MHz): δ, ppm −73.37 (d, 6F, *J =* 716 Hz).

### Methods

#### NMR and Mass Spectroscopy

NMR spectra were recorded with a Bruker 400 MHz NMR spectrometer using CDCl_3_ as the solvent. ESI mass spectra were recorded on a Bruker MicroTOF III mass spectrometer.

#### Electrochemistry

Cyclic and differential pulse voltammetric experiments (CH_3_CN, 0.1 M tetrabutylammonium hexafluorophosphate, (TBA·PF_6_)) were performed on a BAS Epsilon electrochemical analyzer (working electrode: Pt, auxiliary electrodes: Pt wire, reference electrode: Ag wire). The ferrocene couple (*E*_1/2_ (Fc^+^/Fc) = 0.40 V in CH_3_CN, 0.1 M TBA·PF_6_ under our experimental conditions)^50^ was used to calibrate the redox potentials.

#### Optical Spectroscopy

Steady-state UV–visible absorption spectra were recorded with a Cary 100 UV–vis spectrometer. The concentrations of the samples used for these measurements ranged from 10^–6^ M (porphyrin Soret band) to 10^–5^ M (Q-bands) solutions. Steady-state fluorescence spectra were recorded using a Photon Technologies International Quanta Master 8075-11 spectrofluorometer, equipped with a 75 W xenon lamp, running FelixGX software. An excitation wavelength of 550 nm was used, and the optical density (OD) was held constant at 0.2 for all the compounds. The fluorescence lifetimes were evaluated using a Horiba Yvon Nanolog Spectrofluorometer equipped with time-correlated single-photon counting and nanoLED excitation sources. A right-angle detection method was used.

#### Femtosecond and Nanosecond Laser Flash Photolysis

Femtosecond transient studies were performed using an Ultrafast Femtosecond Laser Source (Astrella) by Coherent, which incorporates a diode, mode-locked Ti:Sapphire laser (Vitara), and diode-pumped intracavity doubled Nd:YLF laser (Revolution) to generate a fundamental compressed laser of 800 nm and power output of 5.24 W. A Helios transient absorption spectrometer coupled with a femtosecond harmonics generator, both provided by Ultrafast Systems LLC, will be used for optical detection. The source for the pump pulse is derived from the fundamental output of Astrella (compressed output 5.24 W, pulse width 100 fs, 800 nm at a repetition rate of 1 kHz) by introducing 95% of the beam into the OPA while the other 5% is sent to the delay line and white light-generating crystal. The beam sent through the OPA is termed the pump beam, as it is used to excite the sample. The beam sent through the delay line and crystal is termed the probe beam as it shows what spectral changes occur in the sample with time. The OPA takes the 800 nm fundamental and converts it into a specific wavelength in the 400–2200 nm range, which allows the excitation wavelength to be selected. Kinetic traces at appropriate wavelengths were assembled from the time-resolved spectral data. Data analysis was performed using Surface Xplorer software. All measurements were conducted in degassed solutions at 298 K.

#### Transient EPR Spectroscopy

TREPR spectra were recorded at 80 K on a Bruker Elexsys E580 X-band spectrometer equipped with a dielectric cavity and a CF935 cryostat. The microwave power used for the TREPR experiments was about 0.6 mW. Time/field data sets were recorded in direct-detection mode without field modulation using diode detection; the signal was sampled using the built-in SpecJet digitizer (6 ns per point). The samples were photoexcited at 532 nm by ∼5 ns-long pulses with an energy of ∼4 mJ and a repetition rate of 10 Hz using the second-harmonic of a CONTINUUM Surelite Nd:YAG laser.

#### DFT Calculations

Initial atomic coordinates of SbP-OCH_2_O-SbP were constructed using Avogadro^51^ and the X-ray structure of SbP·PF_6_.^52^ In the structure of SbP, the phenyl groups on opposite sides of the porphyrin ring are in an eclipsed conformation as a result of interactions between neighboring molecules. Initial attempts to optimize the structure of SbP-OCH_2_O-SbP showed that this conformation is unfavorable, and so the phenyl groups were rotated to a staggered conformation. The two porphyrin rings were also rotated to a staggered conformation with respect to one another. A molecular mechanics optimization of this starting structure was performed in Avogadro using the UFF force field.^53^ The structure was then optimized in the singlet ground state using Orca release 6.1.0 ^54–56^ with the CAM-B3LYP functional^57^ and the def2-SVP basis set,^58^ with the D3BJ dispersion correction.^59,60^ Because the triplet-state TREPR spectrum and the absorption spectrum of SbP do not show any apparent influence of the heavy Sb atom, relativistic effects were ignored and the Stuttgart–Dresden effective core potential was used for Sb.^61^ The resolution of identity approximation^62–64^ using the Def2-J auxiliary basis set^65,66^ was used to speed up the calculations. The structure of SbP-OCH_2_O-SbP was also optimized in the lowest triplet state using the optimized structure of the singlet state as a starting point. A restricted open-shell calculation was then carried out on the optimized triplet-state structure to obtain the spin density and molecular orbitals. The structures of the intermediate SbP-OCH_2_Br and CH_2_Br_2_ were optimized in a similar manner, and Hirschfeld population analysis^67^ was carried out to investigate the polarity of their C–Br bonds. The energies and transition densities for the excited singlet states were calculated by time-dependent density functional theory, at the same level of theory as used for the geometry optimization, except that the conductor-like polarizable continuum model^68^ was used to model acetonitrile as the solvent.

## Results and Discussion

### Synthesis

Scheme 1 shows the synthesis of the homodimer SbP-OCH_2_O-SbP·(PF_6_)_2_, which is described in detail in the Experimental Section. The dimer was synthesized starting with SbP-OH·PF_6_ and excess of CH_2_Br_2_ in the presence of K_2_CO_3_. The deprotonation of the axial-OH group results in SbP-O^–^, which undergoes two subsequent O-alkyl substitution reactions with CH_2_Br_2_ to yield the homodimer. Remarkably, the second step of this reaction between SbP-O^–^ and SbP-OCH_2_Br is favored over the reaction between SbP-O^–^ and CH_2_Br_2_ even though the latter is present in a large excess. Two factors likely play a role in determining which of these two reactions dominates. First, SbP-OCH_2_Br carries a positive charge while CH_2_Br_2_ is neutral; thus, the electrostatic attraction between the negatively charged oxygen in SbP-O^–^ and the positive charge in SbP-OCH_2_Br affects the reaction rate between these two species. Second, the strong electron-withdrawing ability of the Sb­(V) center leads to greater polarization of the C–Br bond in SbP-OCH_2_Br compared to CH_2_Br_2_. A Hirschfeld population analysis from DFT calculations bears this out^17^ and predicts that the difference in the local charges across the C–Br bond is 0.20 in SbP-OCH_2_Br but only 0.08 in CH_2_Br_2_. This indicates a much higher ionic character in the C–Br bond, which is known to result in lower activation energy in S_N_2 reactions and is likely the main factor favoring dimer formation.^69^ The synthesis of the reference porphyrin SbP·PF_6_ is reported elsewhere.^52^

### Structural Characterization

ESI mass spectrometry was used to carry out an initial characterization of the investigated compounds (see Figures S1 and S2). The homodimer shows an intense parent ion peak corresponding to the mass (*m*/*z*) of [M – 2PF_6_]^2+^ with matching isotope distribution. The ^1^H, ^13^C, ^19^F, and ^31^P NMR spectra of the investigated porphyrins and their respective monomer compounds are shown in Figures S3–S8. In the ^1^H spectrum of SbP-OCH_2_O-SbP·(PF_6_)_2_, the resonances of the protons on the bridging −OCH_2_O– unit show shielding effects and are strongly shifted upfield to −6.18 ppm due to the ring current effect of the two porphyrin macrocycles. Figure S7 compares the ^1^H NMR spectra of SbP-OH·PF_6_ and homodimer SbP-OCH_2_O-SbP·(PF_6_)_2_. The protons from the homodimer appear at a higher field due to the additional ring current. The ^31^P NMR spectra of all the investigated compounds revealed five lines of a septet at −144.68 ppm from the PF_6_^–^ counterion, as shown in Figures S3 and S5. Careful examination of the ^1^H NMR spectrum of the homodimer reveals broadening of peaks from the *meso*-phenyl protons. To test whether this is the result of motional dynamics, variable temperature ^1^H NMR studies were performed (see Figure S8). With increasing temperature, the peaks broaden and then narrow, which is typical of motional averaging.

### Absorption Spectroscopy

The UV–visible spectra of SbP-OCH_2_O-SbP·(PF_6_)_2_ and its reference compound SbP·PF_6_ in CH_3_CN are shown in Figure 1(top). The band positions (Q-band and B- or Soret band) and their molar extinction coefficients are summarized in Table 1. As shown in Figure 1, the absorption spectrum of the dyad is significantly different from that of its monomer. The monomer shows a sharp Soret band at 418 nm, and two Q-bands at 550 and 590 nm. The Soret band of the homodimer is blue-shifted to 410 nm and is considerably broader than the Soret band of the monomer. In contrast, the Q-bands are red-shifted to 553 and 595 nm. Such a shift has been observed in other strongly coupled porphyrin dimers and attributed to the delocalization of orbitals across the two porphyrin rings.^70^ The observed molar extinction coefficients of the dimer are much higher than those of the monomer, as expected, due to the combination of two SbP units per molecule. The spectrum of the SbP monomer can be described using the four-orbital model first proposed by Gouterman.^71,72^ The model describes the absorptions as linear combinations of one-electron excitations between the occupied a_1u_ and a_2u_ molecular orbitals and the doubly degenerate unoccupied e_g_ orbital. Four transitions are predicted, two of which are *x*-polarized and two of which are *y*-polarized. Each pair of transitions with the same polarization is split into a symmetric and antisymmetric combination of one-electron excitations by the configuration interaction. The transitions to the symmetric combinations are symmetry allowed and form the Soret band, while the forbidden transitions to the antisymmetric combinations form the Q-band. For metallo-porphyrins with 4-fold rotation symmetry, the *x*- and *y*-components are degenerate. Thus, the Soret band and Q-band of SbP are produced by two pairs of degenerate transitions. The Q-band also displays two peaks of a vibronic progression.

**Figure 1:**
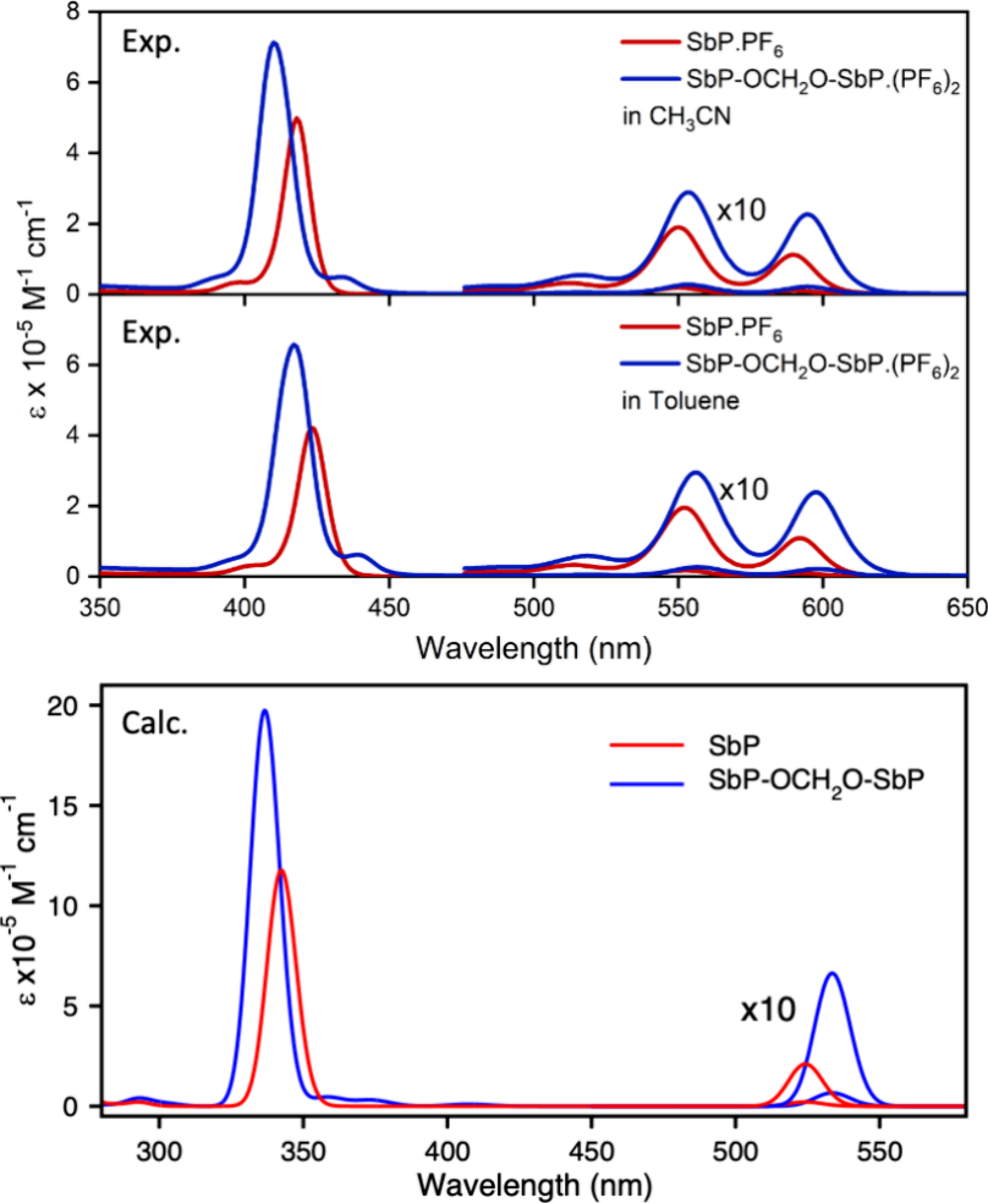
Top: absorption spectra of SbP·PF_6_ (red) and SbP-OCH_2_O-SbP·(PF_6_)_2_ (blue) in CH_3_CN (upper panel) and toluene (lower panel). Bottom: calculated absorption spectra of [SbP-OCH_2_O-SbP]^2+^ and SbP^+^. The spectra were generated using Orca_mapspc by convoluting the calculated vertical excitation energies with a Gaussian line shape and converting the oscillator strengths to extinction coefficients. The line width of the Soret (*B*) bands was set to 150 cm^–1^, and 100 cm^–1^ was used for the Q-bands to roughly match the experimental line widths.

**Table 1:** Optical and Redox Data of Investigated Compounds in CH_3_CN

	potential [V vs SCE]		
sample	oxidation	reduction	absorptionλ_max_ [nm] (log (ε [M^–1^ cm^–1^]))
SbP·PF_6_	1.96	–0.37, – 0.80	418 (5.70), 550 (4.28), 590 (4.05)
SbP-OCH_2_O-SbP·(PF_6_)_2_	1.80	–0.35, – 0.79, – 0.91	410 (5.85), 553 (4.46), 595 (4.35)

For chromophore dimers, the point dipole model of exciton coupling, first formulated by Kasha,^73^ predicts that the upper electronic state of an electronic transition is split into two states by the interaction between the transition dipoles of the two chromophores. If the interaction is stronger than the energy differences between the excited states of the individual chromophores, then the two states of the dimer are symmetric and antisymmetric combinations of the monomer states. When the angle between the transition dipoles of the two chromophores is small, the symmetric state is higher in energy, and the corresponding transition to it is symmetry allowed, while the lower-energy transition to the antisymmetric state is forbidden, so that a strong, blue-shifted and weak red-shifted peaks are observed. The behavior of the Soret band in SbP-OCH_2_O-SbP·(PF_6_)_2_ is in good qualitative agreement with this model and displays these characteristic shifts and intensities. Similar behavior has been observed in other porphyrin dimer systems.^74–13^ For the Q-bands, the point dipole exciton coupling between the porphyrins is expected to be weaker because they have small transition dipoles, and the observed shifts are very small in most cases.^74^ Thus, the observed 3–5 nm red shift of the Q-bands in SbP-OCH_2_O-SbP·(PF_6_)_2_ is unusual and suggests that the mixing of the electronic wave functions of the two porphyrins is stronger than that described by dipole–dipole exciton coupling.

### DFT Ground-State Properties

Figure 2 shows the optimized structure of the singlet ground state (SbP-OCH_2_O-SbP)^2+^. The computational details are given in the Experimental Section. The optimized structure shows that the two porphyrin rings are nearly parallel, with an angle of ∼15 ° between them, at a distance of 5.85 Å between the two Sb centers. This distance is similar to but slightly shorter than the Mg–Mg distances 6.3 and 8.2 Å, respectively, in the chlorophyl dimers P_700_ and P_680_ of photosystems I and II. The two rings are rotated with respect to one another, so that the phenyl groups are in a staggered conformation. The dimerization of the porphyrins does not result in any significant distortion of the planarity of the porphyrin rings, and the Sb centers lie in the respective porphyrin planes. The optimized structure of the monomer SbP^+^ is shown in Figure S9, along with the frontier orbitals corresponding to Gouterman’s four-orbital model. The corresponding eight frontier orbitals of the dimer are shown in the bottom panel of Figure 2. The HOMO and HOMO–1 of the dimer are the a_2u_ HOMO orbitals of the two constituent monomers and are localized on a single porphyrin, while the HOMO–2 and HOMO–3 are symmetric and antisymmetric combinations of the monomer a_1u_ orbitals and are delocalized over both porphyrins. The LUMO, LUMO+1, LUMO+2, and LUMO+3 are linear combinations of the unoccupied e_g_ orbitals of the monomers and are also delocalized.

**Figure 2:**
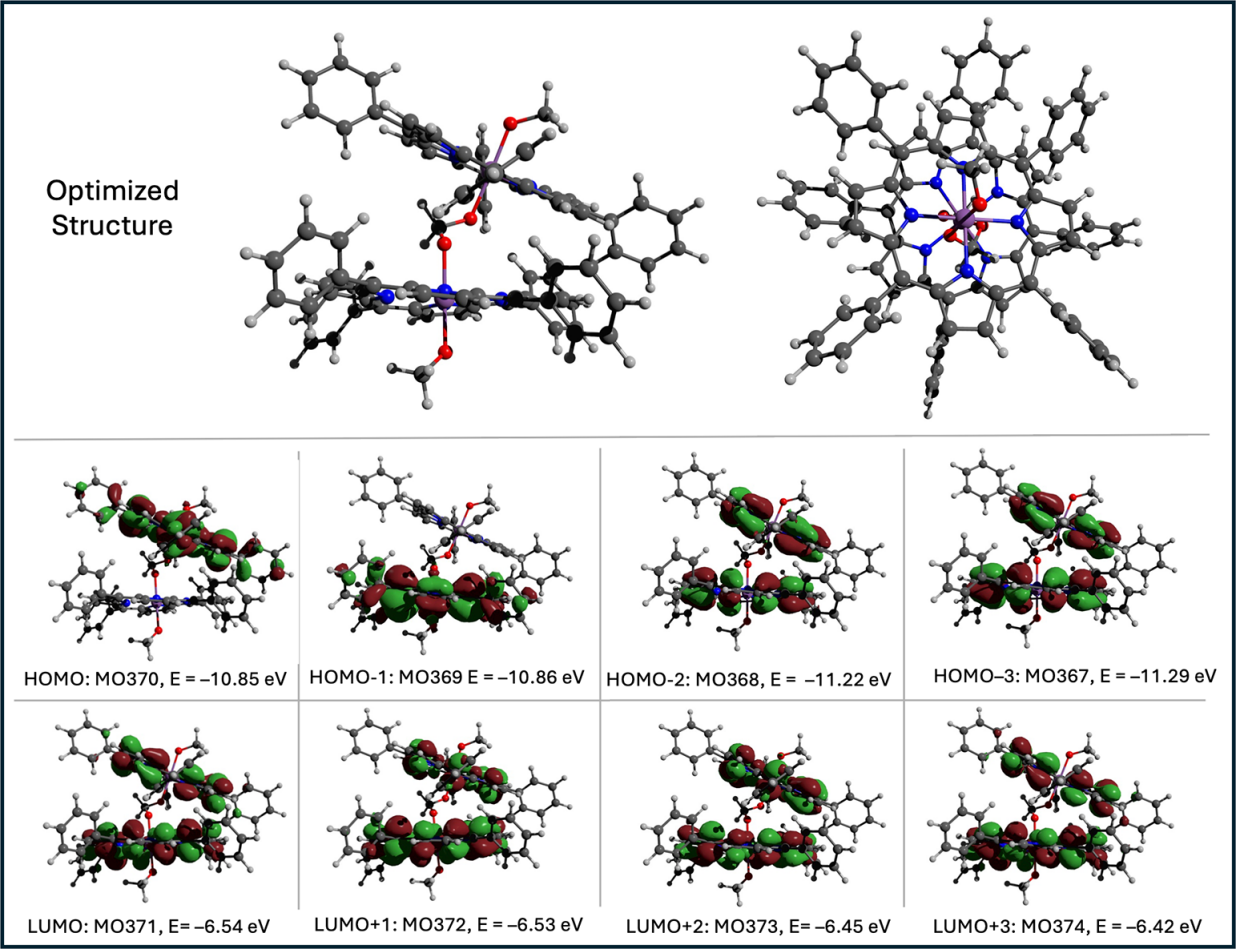
DFT-optimized structure and the frontier molecular orbitals of SbP-OCH_2_O-SbP·(PF_6_)_2_.

### DFT Excited-State Properties

Figure 1 bottom panel and Figure S10 show the absorption spectra of SbP^+^ and (SbP-OCH_2_O-SbP)^2+^ calculated by time-dependent density functional theory (TDDFT) using the CAM-B3LYP functional and the def2-SVP basis set. The computed spectra show a strong Soret band and a weak Q-band; however, because the calculation does not include vibronic states, it yields only vertical excitation energies and does not reveal the vibronic structure of the Q-band. For both the monomer and dimer, the calculation overestimates the Soret band energies by ∼4500 cm^–1^ and the Q-band energy by about 2000 cm^–1^. Despite this systematic error in the energies, the higher absorbance of the dimer compared to the monomer and the blue shift of the Soret band and red shift of the Q-band are reproduced. The transition densities for the four *Q*-band and four Soret (*B*) band transitions are plotted in Figure 3 and clearly show that both porphyrins are excited in all eight transitions as expected for coupled chromophores.

**Figure 3:**
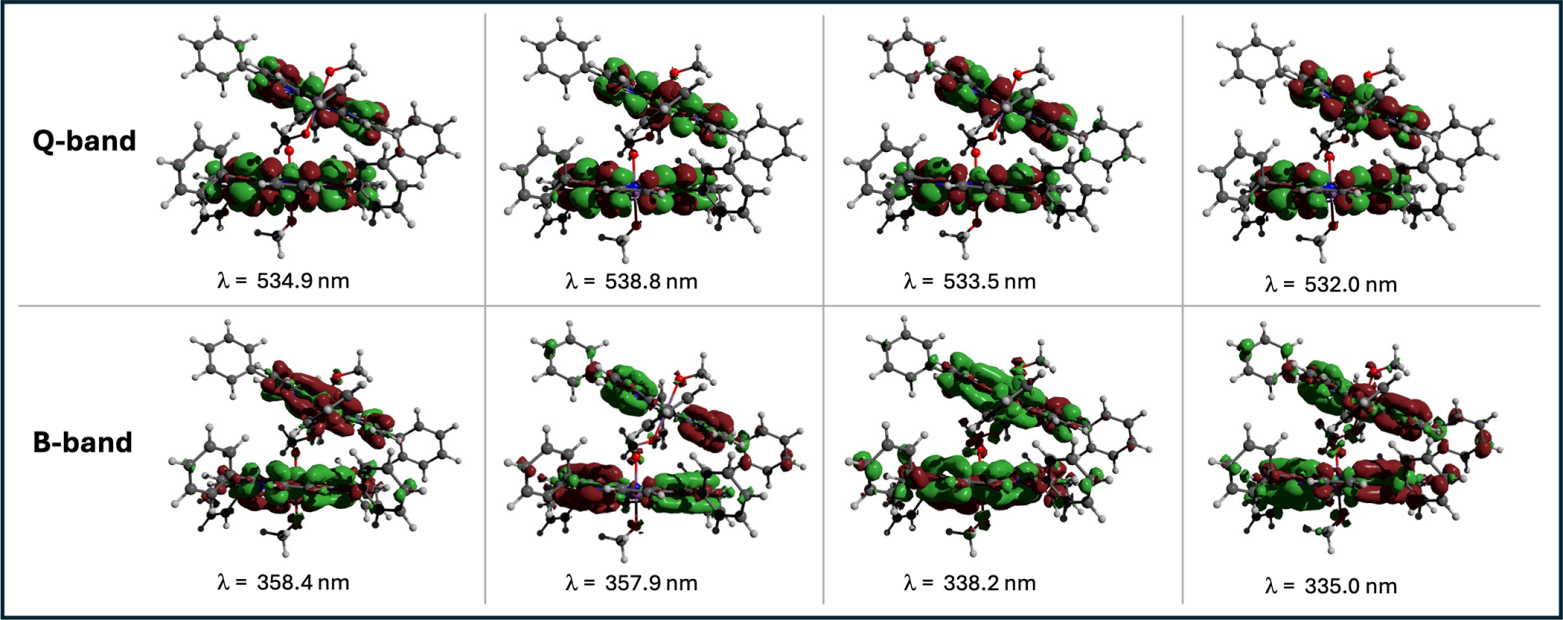
Transition densities of the main bands in the absorption spectrum of [SbP-OCH_2_O-SbP]^2+^ from time-dependent DFT calculations.

### Electrochemistry

Cyclic voltammograms of SbP-OCH_2_O-SbP·(PF_6_)_2_ and the reference compound SbP·PF_6_ was measured in CH_3_CN with 0.1 M TBA·PF_6_ and ferrocene as an internal standard. Representative voltammograms are shown in Figure 4, and the data are summarized in Table 1. The redox processes of all the compounds are found to be one-electron reversible based on the peak-to-peak separation values and the cathodic-to-anodic peak current ratio. The voltammogram of SbP·PF_6_ revealed two reductions at −0.37 and – 0.80 V and one oxidation process at 1.80 V. The anodic scan of the dimer SbP-OCH_2_O-SbP·(PF_6_)_2_ revealed the same oxidation process at 1.80 V. This is consistent with the DFT studies that indicate that the HOMO is localized on one of the two SbP units (see Figure 2). The cathodic scan reveals three reduction processes at −0.35, −0.80, and −0.91 V. The first of these occurs at almost the same potential as the first reduction of the monomer SbP·PF_6_, while the second reduction peak is split. The first of these is at the same potential (−0.80 V) as in the monomer, but the second is shifted more negatively by 0.11 V. This shift is likely due to the electrostatic repulsion between the two doubly reduced porphyrins in the dimer. The fact that this effect is larger for the reduction of the dimer compared to oxidation is consistent with the greater delocalization of the LUMO and LUMO+1 compared to the HOMO and HOMO–1 (Figure 2).

**Figure 4:**
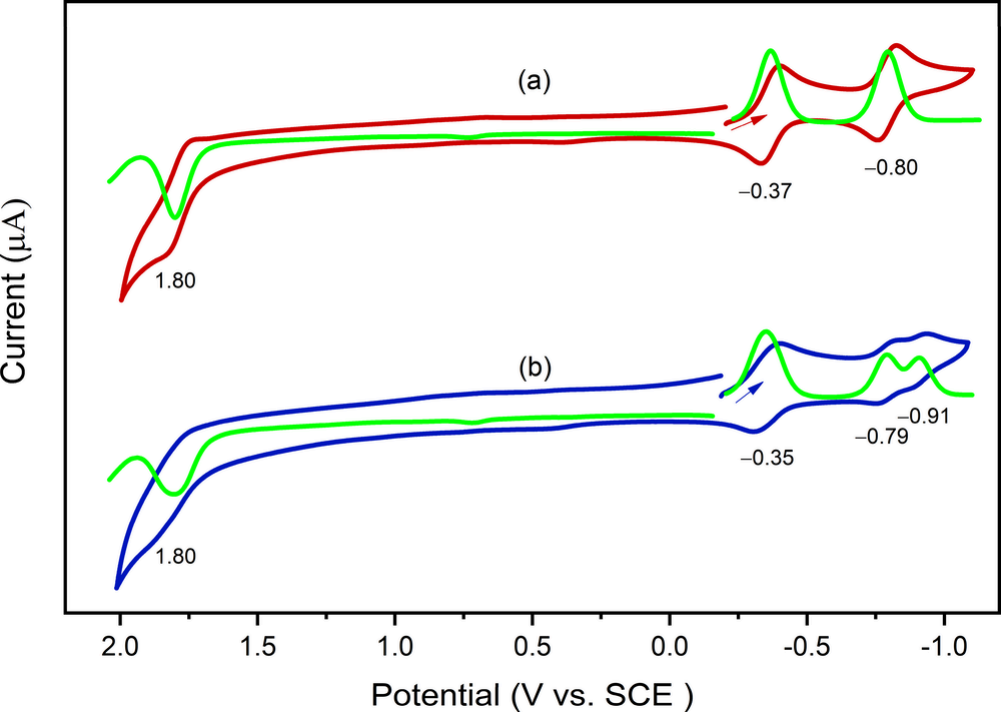
Cyclic and differential voltammogram of (a) SbP·PF_6_ and (b) SbP-OCH_2_O-SbP·(PF_6_)_2_. The experiments were performed in CH_3_CN with 0.1 M TBA·PF_6_. Scan rate 100 mV/s.

### Emission Spectroscopy

Steady-state fluorescence of the homodimer SbP-OCH_2_O-SbP·(PF_6_)_2_ and its reference compound SbP·PF_6_ was measured in polar CH_3_CN and nonpolar toluene with excitation wavelengths of 416 and 550 nm, respectively. Figure 5 shows the fluorescence spectra, and Table 2 summarizes the emission maxima and quantum yields (Φ) in toluene and CH_3_CN. As can be seen in Figure 5a, at the excitation at 416 or 550 nm, the reference compound SbP·PF_6_ exhibits two fluorescence bands at ∼600 and ∼654 nm in CH_3_CN, respectively. A similar two-band spectrum was observed from the homodimer SbP-OCH_2_O-SbP·(PF_6_)_2_. However, the band positions are red-shifted to ∼608 and ∼662, and the intensity or Φ decreases slightly. Similar results were observed in toluene solutions (see Figure 5b). These results suggest that the fluorescence originates from the lowest excited singlet state in both monomer and dimer. The red shift in the dimer suggests that this state is slightly lower in energy than in the monomer. The lower fluorescence intensity in the dimer indicates that the relaxation process from the lowest excited state is altered in the dimer molecule.

**Figure 5:**
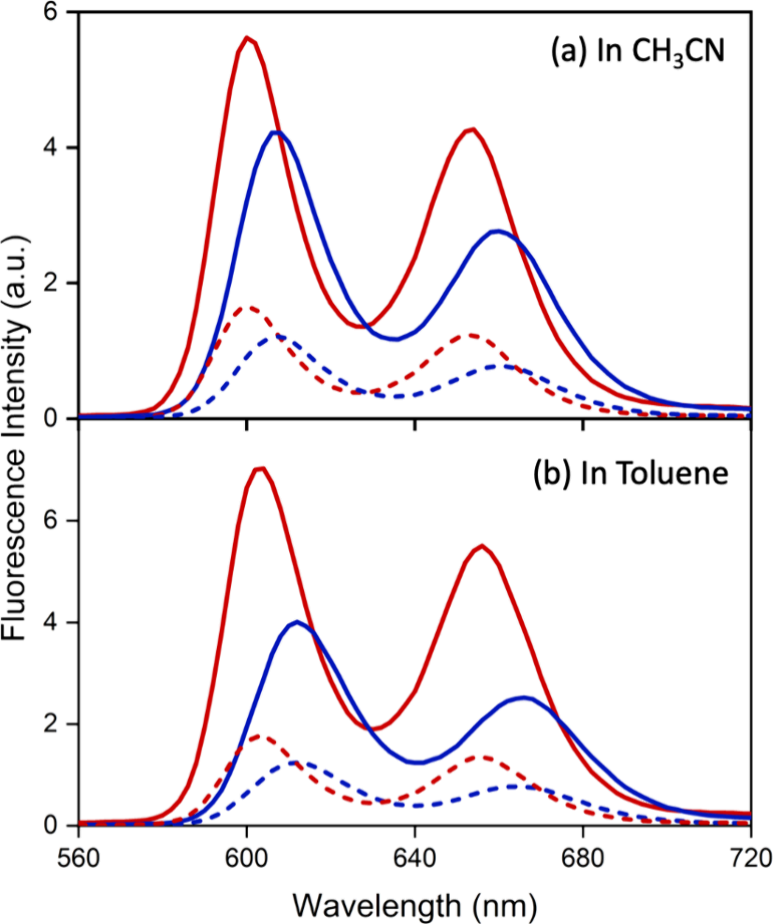
Fluorescence spectra of SbP·PF_6_ (red) and SbP-OCH_2_O-SbP·(PF_6_)_2_ (blue) in CH_3_CN (top) and toluene (bottom). Solid and dashed curves represent the excitation at 416 and 550 nm, respectively. Samples were maintained equal optical densities to make the comparison valid.

**Table 2:** TCSPC Lifetimes of Fluorescence Emission

		average lifetime (ns)^a^		
sample	solvent	τ_1_ (% contribution)	τ_2_ (% contribution)	fluorescence^b^ λ_max_, nm(Φ)
SbP·PF_6_	CH_3_CN	1.36 (100%)		600, 654(0.040)
	toluene	1.23 (100%)		604, 656(0.045)
SbP-OCH_2_O-SbP·(PF_6_)_2_	CH_3_CN	1.31(100%)		608, 662(0.034)
	toluene	1.05 (87%)	3.13 (13%)	612, 664(0.038)

a Excitation at 560 nm.

b Excitation at 550 nm.

To determine the fluorescence lifetimes, time-resolved fluorescence studies of monomer and dimer were carried out in CH_3_CN and toluene. The samples were excited at 560 nm, and the emission from the high-energy fluorescence band at 600 nm was collected. The representative fluorescence decay profiles are illustrated in Figure S11, and the lifetime data are summarized in Table 2. All the decay profiles were found to be monoexponential except for that of SbP-OCH_2_O-SbP·(PF_6_)_2_ in toluene, which is biexponential with a long-lived minor component. The monomer decays with lifetimes of 1.36 and 1.23 ns, whereas the homodimer decays with slightly faster with lifetimes of 1.31 and 1.05 ns in CH_3_CN and toluene, respectively. These observations indicated a weak exciton interaction in the cofacial homodimer, which resulted in the weak perturbation in excited singlet state.^13,76–78^ Overall, the lifetime trends are complementary to the steady-state fluorescence data.

### Transient Absorption Studies

Femtosecond transient absorption (*fs*-TA) spectral studies were also performed in CH_3_CN and toluene, at an excitation wavelength of 410 nm (corresponding to the Soret band) to obtain more information about the excited-state dynamics and the presence of exciton coupling in the dimer.

Figure 6a shows the *fs*-TA spectra of SbP-OCH_2_O-SbP·(PF_6_)_2_ at the indicated delay time in CH_3_CN. The S_2_ → S_1_ internal conversion, followed by the solvent relaxation of the S_1_ state, occurred within a few ps. The S_1_ state was characterized by excited-state absorption (ESA) peaks at 450, 570, 625, and 1220 nm. The 1220 nm peak in the near-IR region corresponds to the S_1_→S_2_ excited-state absorption and is observed for both the dimer SbP-OCH_2_O-SbP·(PF_6_)_2_ and the monomer SbP·PF_6_ (Figure 6b). However, in the dimer, it is broad and has a shorter lifetime of 271.3 ps, compared to the monomer peak lifetime of 900.1 ps (see Figure 6c). This difference has been attributed to the presence of exciton coupling between the two porphyrin units of the dimer.^79^ In addition to the ESA peaks, some negative peaks are also present at 550, 595, and 655 nm. By comparison with the absorption and emission spectra, the peaks at 550 and 595 nm are assigned to ground-state bleaching (GSB) of the *Q*-bands, and the peak at 650 nm is assigned to stimulated emission (SE). The decay and recovery of the positive and negative peaks respectively are accompanied by the rise of new peaks at 492, 690, and 835 nm (see Figure 6c at longer delay), as a result of population of the triplet state T_1_ *via* intersystem crossing (ISC). To analyze the *fs*-TA spectral data, target analysis was carried out on the visible region of the data set using a three-component decay scheme representing S_2_ → S_1_ → T_1_ with the lifetimes of 1.2 ps, 1.3 ns, and >3 ns, respectively. The decay-associated spectra (DAS) obtained from the analysis are shown in Figure 7. The lifetime of the S_1_ state corresponds well with the fluorescence lifetime (see Table 2), and the nondecaying component (>3 ns) is consistent with the lifetime of the triplet state (8.54 μs) obtained from nanosecond transient absorption (*ns*-TA) measurements (see Figure S12a). The DAS of the dimer SbP-OCH_2_O-SbP·(PF_6_)_2_ in CH_3_CN is shown in Figure 7a. The corresponding analysis of the monomer SbP·PF_6_ in CH_3_CN (Figure 7b) yields lifetimes of the S_2_, S_1_, and T_1_ excited states as 9.5 ps, 998.6 ps, and >3 ns (3.40 μs from *ns*-TA spectra, Figure S12b), respectively.

**Figure 6:**
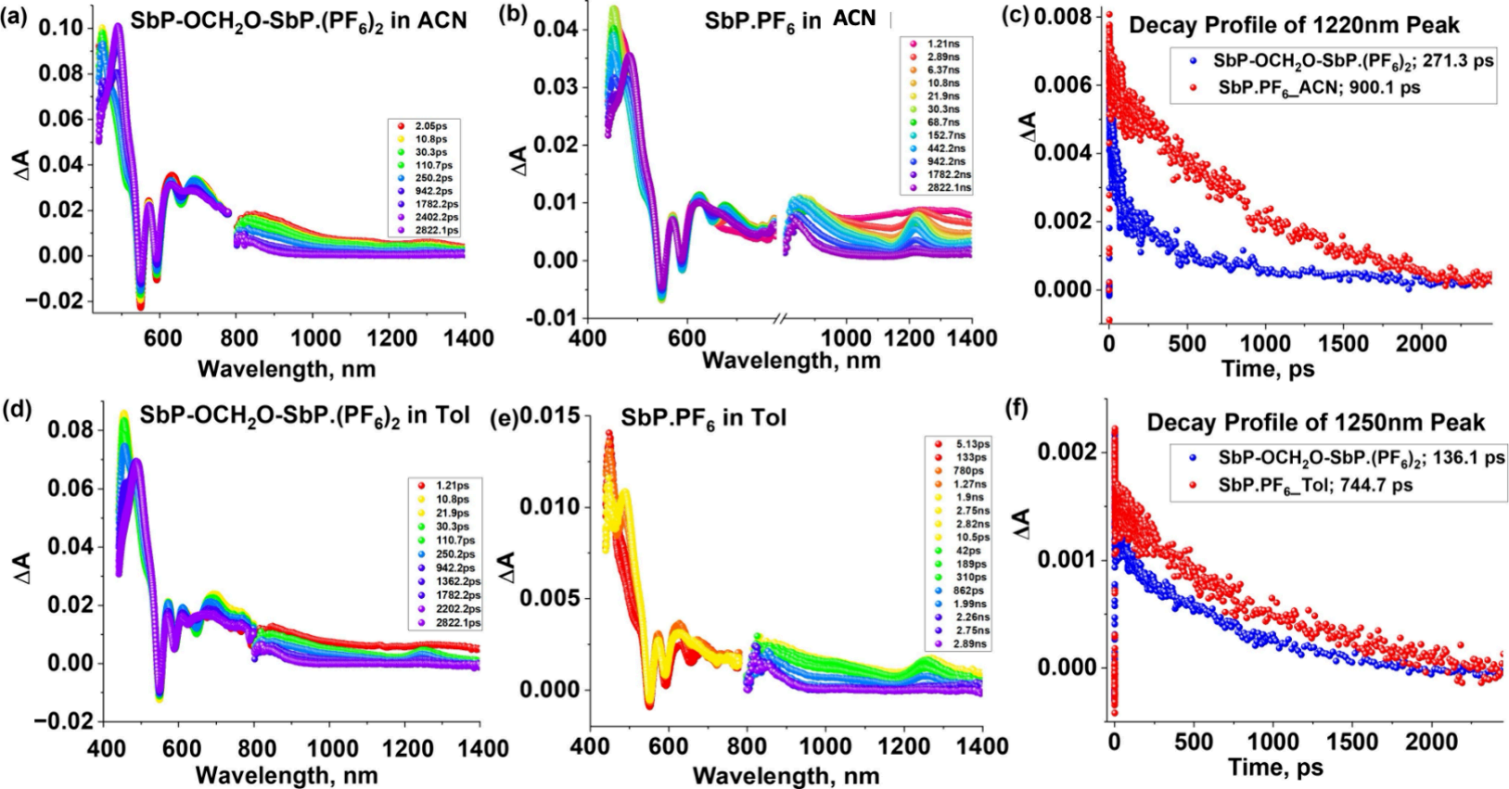
*fs*-TA spectra at the indicated delay times of SbP-OCH_2_O-SbP·(PF_6_)_2_ in CH_3_CN (ACN) and toluene (Tol) (a) and (d), respectively, while for SbP·PF_6_, (b) is in CH_3_CN and (d) is in toluene. (c) and (f) show the decay profile of the 1220 nm peak in acetonitrile and the 1250 nm peak of SbP-OCH_2_O-SbP·(PF_6_)_2_ and SbP·PF_6_ with their lifetimes in the legends. Excitation wavelength = 410 nm.

**Figure 7:**
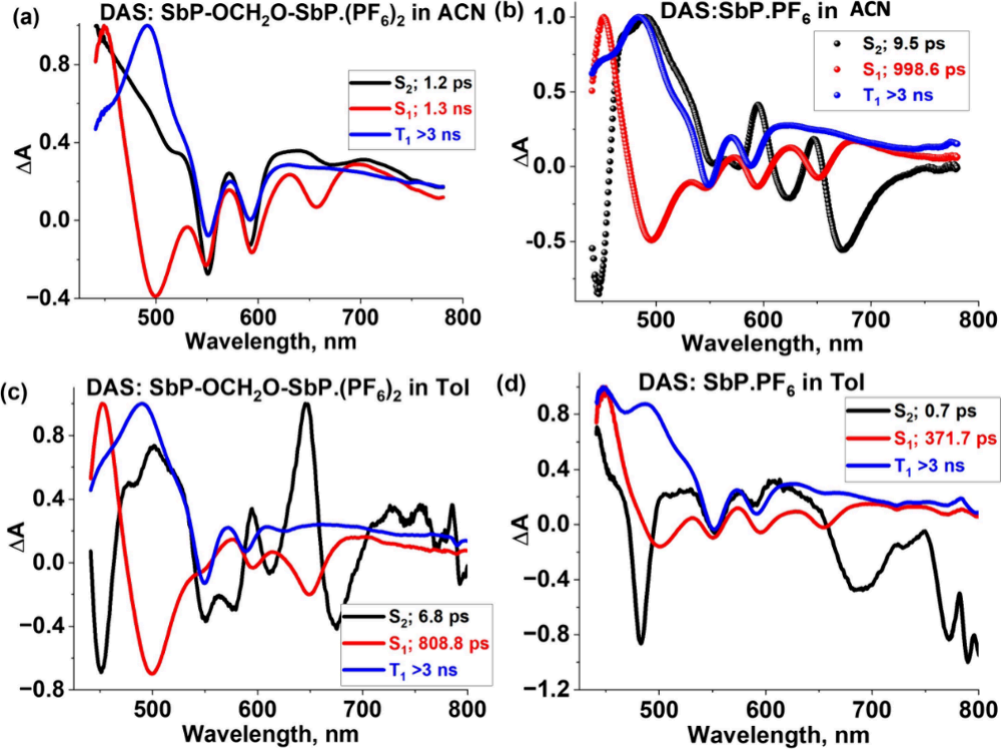
Decay-associated spectra (DAS) of SbP-OCH_2_O-SbP·(PF_6_)_2_ and SbP·PF_6_ in CH_3_CN (a, b) and in Toluene (c, d), respectively.

Similar studies were performed in toluene to investigate the effect of solvent polarity on exciton coupling between the porphyrin rings. Figure 6d and Figure 6e show the femtosecond transient spectra of the dimer SbP-OCH_2_O-SbP·(PF_6_)_2_ and monomer SbP·PF_6_ in toluene, respectively. The spectral features are similar to those observed in CH_3_CN. That is, ESA peaks at 455, 570, 615, and 1250 nm. In addition, negative peaks at 545, 590, and 650 nm related to GSB and SE were observed. Decay and recovery of positive and negative peaks were accompanied by new peaks at 490, 695, and 840 nm as a result of decay from the S_1_ state to the T_1_ state *via* ISC. The target analysis (see Figure 7c) gave lifetimes of 6.8 ps for the S_2_ state, 808.8 ps for the S_1_ state, and >3 ns for the T_1_ state. The lifetime of the S_1_ state matches well with the TCSPC lifetime (see Table 2), and that of the T_1_ state is consistent with the value of 3.4 μs obtained from *ns*-TA measurements. The decay profile of the triplet state is shown in the inset of Figure S12c. The peak broadening and faster decay of the ESA peak at 1250 nm for the dimer with the lifetime of 136.1 ps confirms the exciton coupling in SbP-OCH_2_O-SbP·(PF_6_)_2_, while the lifetime of the 1250 nm peak of SbP·PF_6_ was 744.7 ps (Figure 6f). The target analysis of SbP·PF_6_ in toluene (see Figure 7d) provides the lifetimes of 0.7 ps, 371.7 ps, and >3 ns for the lifetimes of the S_2_, S_1_, and T_1_ states, respectively. Consistent with the target analysis, the *ns*-TA measurement gave a T_1_ state lifetime of 9.63 μs (Figure S12d).

### Time-Resolved Electron Paramagnetic Studies

The interaction between the two porphyrins of the dimer can also be investigated in the triplet state using transient electron paramagnetic resonance (TREPR) spectroscopy. Porphyrin triplet-state TREPR spectra are dominated by the dipole–dipole zero-field splitting (ZFS) and are sensitive to the distribution of the two unpaired electrons. The ZFS is described by parameters *D* and *E*, which can be extracted from the TREPR spectra. The parameter *D* depends on the deviation of the spin density distribution from spherical symmetry and the average separation of the two unpaired electrons. A highly localized distribution, with high asymmetry, results in a large value of *D*, while a delocalized distribution with nearly spherical symmetry gives a small value of *D*. The parameter *E* describes the deviation of the ZFS tensor from axial symmetry. Thus, for a porphyrin dimer, delocalization of the spin density over both porphyrins would result in a reduction of *D*. Triplet hopping between two sites can also occur in such systems and results in an averaging of the ZFS tensors in the two sites. Its effect on the observed spectra depends on the hopping rate and the relative orientations of the two tensors. Figure 8 shows a comparison of the TREPR spectra of SbP·PF_6_ and SbP-OCH_2_O-SbP·(PF_6_)_2_ measured in MeTHF at 80 K (black spectra) and their simulations (red spectra). The method used to simulate the spectra and the parameters obtained are given in the Supporting Information (SI). The overall width of the spectra is equal to 2D, and as can be seen, there is very little difference between the monomer and dimer, indicating very little delocalization of the spin density. The spectra are also spin polarized because of the selective population of the triplet sublevels during intersystem crossing. Again, the polarization patterns are very similar, indicating that the ISC process is not significantly affected by the dimer formation, consistent with a triplet state localized on a single porphyrin. The most notable difference between the two spectra is that the emission/absorption feature in the middle is more pronounced in the dimer. The simulations show that this is due to a slightly smaller value of *E* in the dimer.

**Figure 8:**
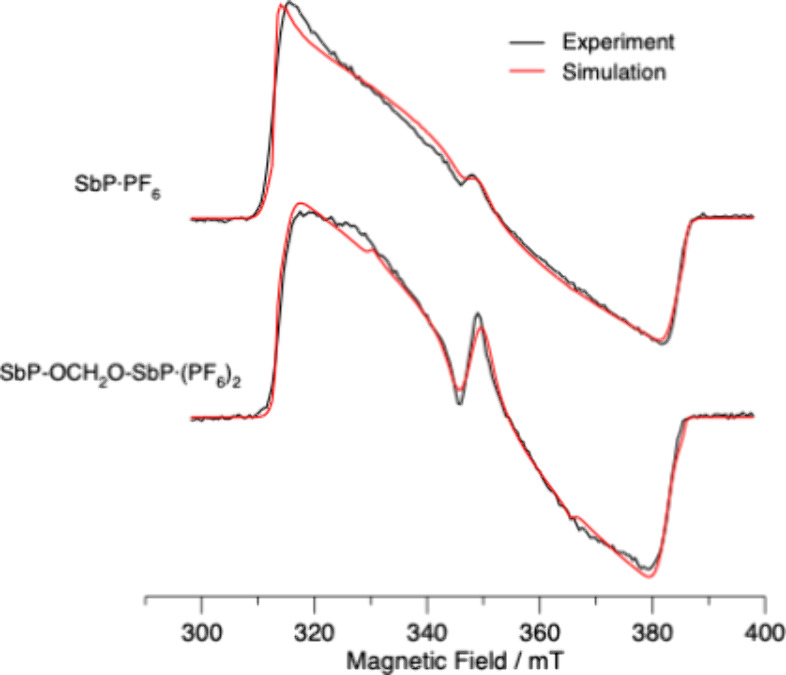
Spin-polarized transient EPR spectra of SbP·PF_6_ and SbP-OCH_2_O-SbP·(PF_6_)_2_ in MeTHF at 80 K. The experimental spectra (black) were extracted from the full time/field data set using a 300 ns-wide window centered at 750 ns after the laser flash and are normalized to the same maximum amplitude. Details of the simulations (red spectra) and the parameters obtained are given in the SI.

The comparison of the TREPR spectra indicates that, in contrast to the excited singlet states, the triplet state is not affected significantly by the interaction between the porphyrins. This is consistent with the expected dependence of the excitonic coupling on the transition dipoles and differences in the mixing of the molecular orbitals. Since excitation from the ground state to the lowest excited triplet state is spin-forbidden, its transition dipole is essentially zero, so that in the dipole–dipole model of excitonic coupling, the triplet state is expected to remain localized. Consistent with the weaker mixing expected in the molecular orbital model, DFT calculations of the lowest triplet state show that the spin density and the frontier orbitals are not delocalized onto both porphyrins (see Figure 9).

**Figure 9:**
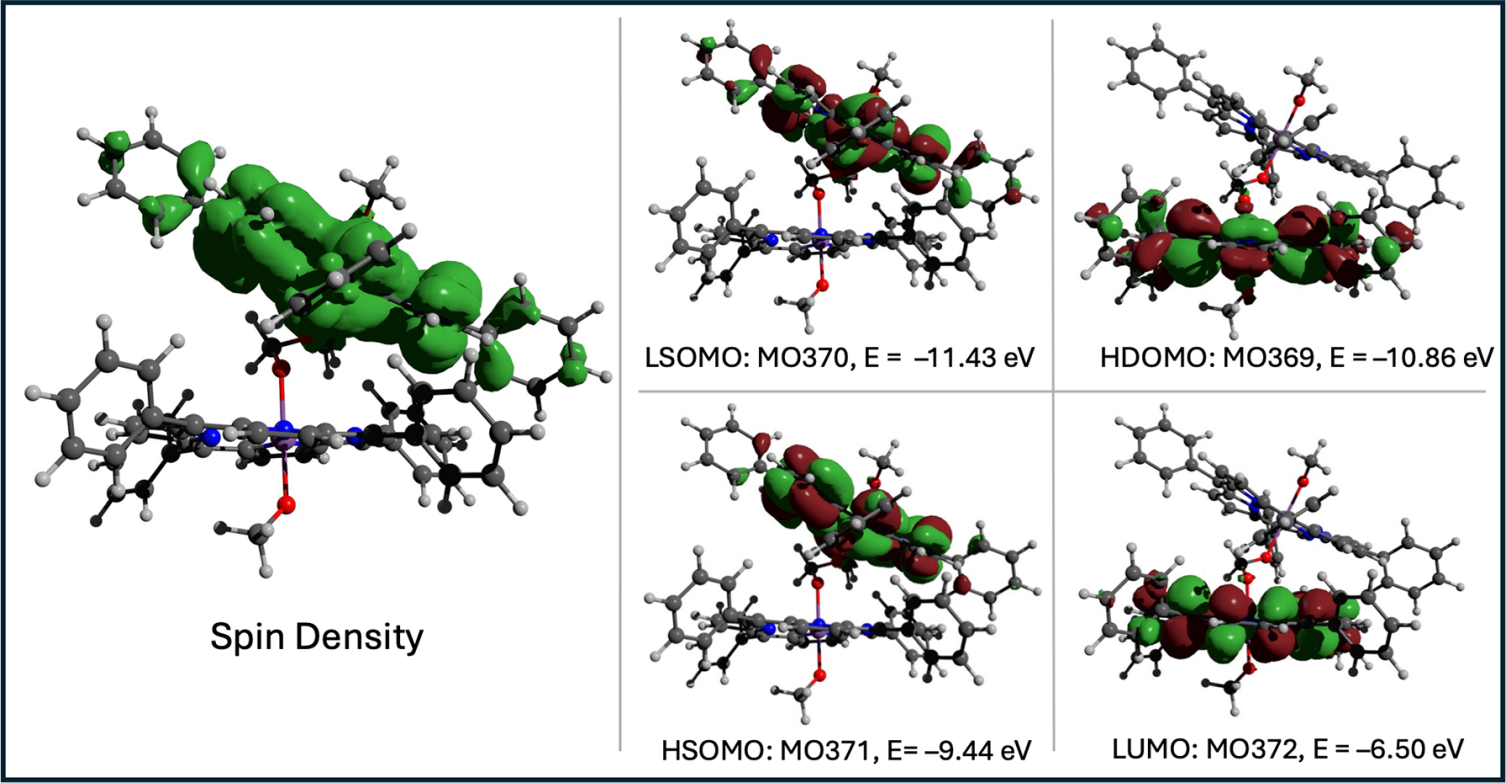
Electron spin density and frontier molecular orbitals of the lowest triplet state of [SbP-OCH_2_O-SbP]^2+^.

### H-Type Cofacial Interactions

According to Kasha’s dipole–dipole theory, upon excitation, the Coulomb coupling (*J*) in a porphyrin dimer results in the formation of two delocalized excited states separated by an energy of 2*J*.^80–82^ The two states consist of in- and out-of-phase linear combinations of the two local excited states. The in-phase combination (*J* > 0) is characterized by an enhanced transition dipole moment relative to the monomer, whereas the out-of-phase (*J* < 0) state is optically dark due to a cancellation of the transition dipole moments. As a result, in H-type cofacial dimers, the *S*- and *Q*-states split into two exciton states, with symmetric higher-energy and antisymmetric lower-energy states. The antisymmetric exciton states are forbidden for transitions from the ground state, whereas the symmetric states are allowed, leading to a blue-shifted absorption band in H-type dimers.

Figure 10 summarizes the energy levels of the investigated compounds in CH_3_CN. The energies of the excited singlet states (^1^SbP* and ^1^SbP**) were calculated from absorption and fluorescence data (Figure S13). The triplet-state energies were obtained from the positions of the phosphorescence bands in the emission spectra (see Figure S14). The optical and redox properties, as well as DFT calculations, indicate differences between the dimer and the corresponding monomer. The absorption spectrum of the dimer shows blue and red shifts of the Soret and *Q*-bands, respectively. A blue-shifted Soret absorption is consistent with Kasha’s theory, but the size of the shift is proportional to the transition dipole. However, *Q*-bands are forbidden transitions with small transition dipoles and this model predicts little or no shift for the *Q*-bands, which is not consistent with the observed red shift. Similar behavior has been observed in strongly coupled porphyrin sandwich compounds and can be rationalized as a result of non-negligible overlap of the porphyrin orbitals, which leads to mixing and delocalization of the MOs over both porphyrins.^70^ Consistent with this picture, the DFT calculations show that the frontier orbitals involved in the Soret and *Q*-bands are delocalized (Figure 2).

**Figure 10:**
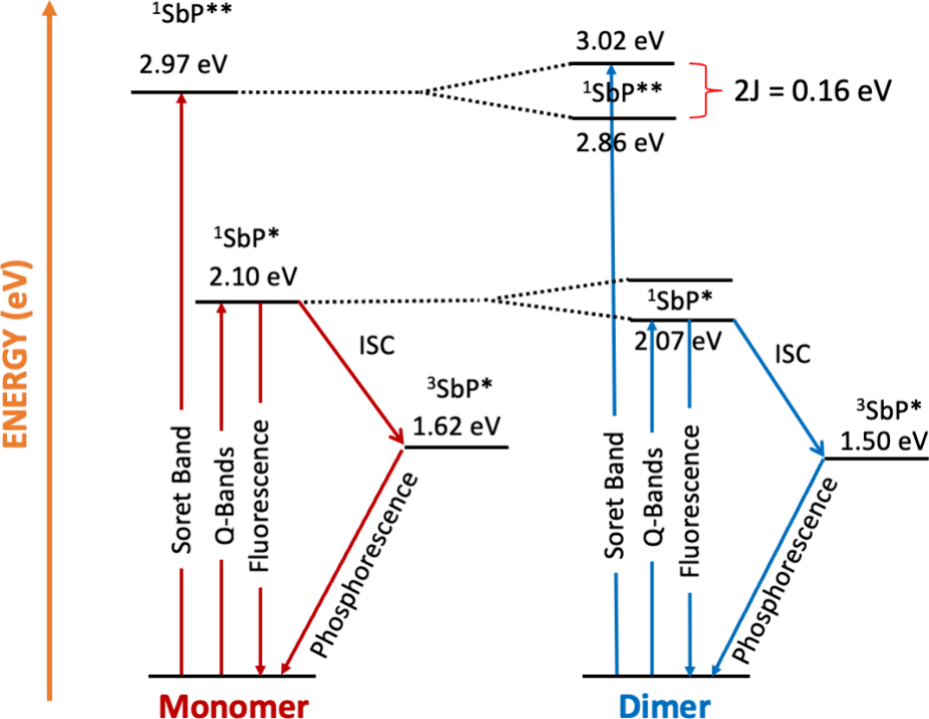
Energy diagram depicting the photo events in CH_3_CN of the studied antimony­(V) porphyrin monomer and the dimer.

In addition to absorption spectral shifts, Kasha also demonstrated that the radiative decay rate is significantly different for monomers and H-type dimers, as rapid relaxation occurs after absorption, thereby efficiently populating the lowest energy state. Because the out-of-phase alignment of transition dipoles characterizes the state, it has no direct radiative coupling to the ground state. The transition is strictly forbidden by symmetry, which should result in the dimer not fluorescing. However, the studied dimer exhibits fluorescence that is only slightly lower than that of the monomer (Figure 5). Many such examples deviate from Kasha’s theory and exhibit fluorescence emission with relatively high quantum yields.^83^ One possible reason for this deviation is the involvement of vibrational modes, which alter the overall symmetry of the lowest excited state and partially allow radiative decay. Another possibility is low efficiency of the competing nonradiative pathways. Hence, one can have strongly emissive H-dimers if the weak radiative decay rate dominates an even weaker nonradiative decay rate. Moreover, Kasha’s theory predicts enhanced phosphorescence because fluorescence from the lowest excited state is suppressed, thereby favoring intersystem crossing to the triplet state and phosphorescence. In the present study, none of these effects were observed; the dimer exhibits considerable fluorescence, and its phosphorescence is lower than that of the monomer. These discrepancies indicate that the point dipole model of the interaction between the two porphyrins is not strictly valid in this case. This is not surprising given that the distance between the two macrocycles (5.85 Å) is smaller than their diameter (∼8 Å). With this close interaction between the porphyrin, the dimer acts more like a single chromophore with molecular orbitals and electronic transitions that are delocalized over the whole complex rather than two isolated chromophores with electronic states that are only slightly perturbed by the dipole–dipole coupling.

## Conclusions

The unique asymmetric axial-bonding ability of hypervalent antimony­(V) porphyrin has been exploited to construct the studied homodimer. Despite the positive charge on each porphyrin unit, the resulting dimer is highly structurally stable under all experimental conditions. The steady-state and redox chemistry both indicate the electronic interactions between cofacially linked porphyrin units, and the spectral trends establish the H-type exciton coupling in the homodimer. Based on its redox properties, the dimer exhibits an unusually high oxidation potential that closely resembles that of the special pair in natural systems. We are currently exploring the possibility of connecting either an electron donor or an acceptor to one of the SbP molecules to initiate charge separation, thereby delocalizing the charge on the dimer to model the reaction center complex for artificial photosynthesis.

## Supplementary Material



## Notes

ESI mass spectra, NMR data, frontier orbitals of monomer, spectral overlaps to calculate *E*_0–0_, phosphorescence spectra, fluorescence lifetime decay profiles, DAS spectra from GloTarAn analysis, ns-TA spectra with triplet-state decay profiles, and TREPR simulation details (PDF)
