# Supplementary material for: Electronically Coupled, Cofacially Linked, Hypervalent Antimony(V) Porphyrin Homodimer: Synthesis, Spectroscopy, and Photochemistry
Source: Inorg Chem. 2026 Mar 24;65(13):7257–68. doi: 10.1021/acs.inorgchem.5c05959 (PMC13058881; doi:10.1021/acs.inorgchem.5c05959)
Supplement: Supplementary file 1 [file ic5c05959_si_001.pdf]

Electronically Coupled, Co-facially Linked, Hypervalent Antimony(V) Porphyrin Homodimer: Synthesis, Spectroscopy, and Photochemistry

Prashanth K. Poddutoori,[a,] Peyton Ellis,[a] Jatan K. Sharma,[b] Niloofar Zarrabi,[a] Art van der Est,[c,*] Francis D'Souza[b,*]*

[a]Department of Chemistry & Biochemistry, University of Minnesota Duluth, 1038 University Drive, Duluth, Minnesota 55812, USA. [b]Department of Chemistry, University of North Texas, 1155 Union Circle, # 305070, Denton, Texas 76203-5017, USA. [c]Department of Chemistry, Brock University, 1812 Sir Isaac Brock Way, St. Catharines, ON, L2S 3A1, Canada.

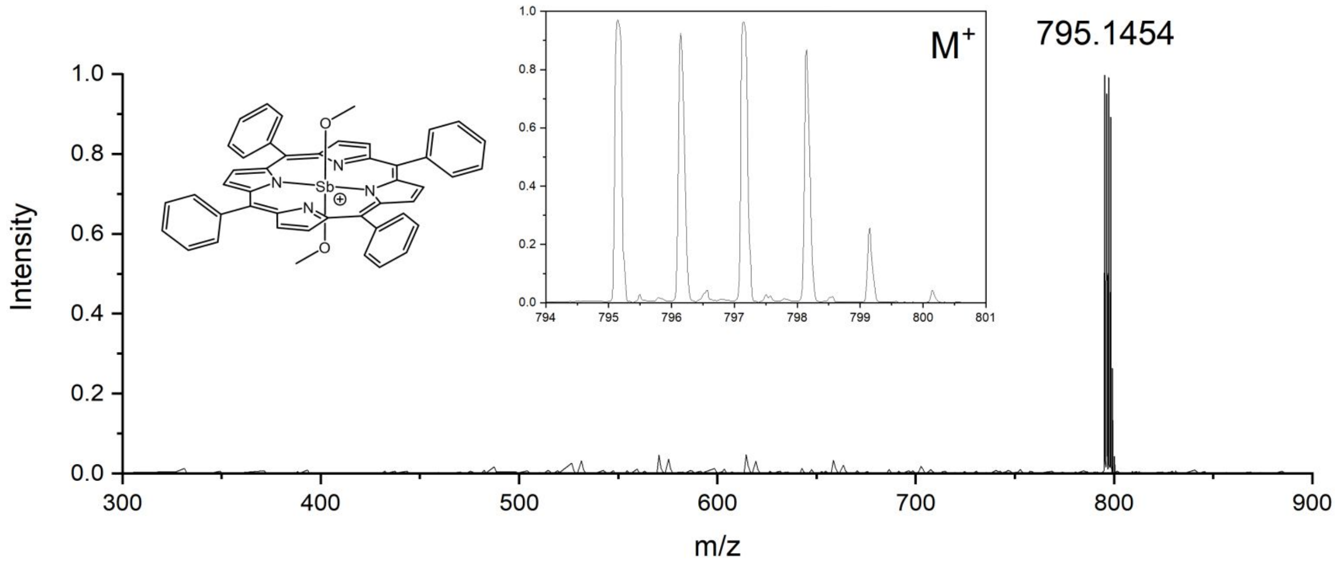

Figure S1. High resolution ESI mass spectra of the SbP·PF_6.

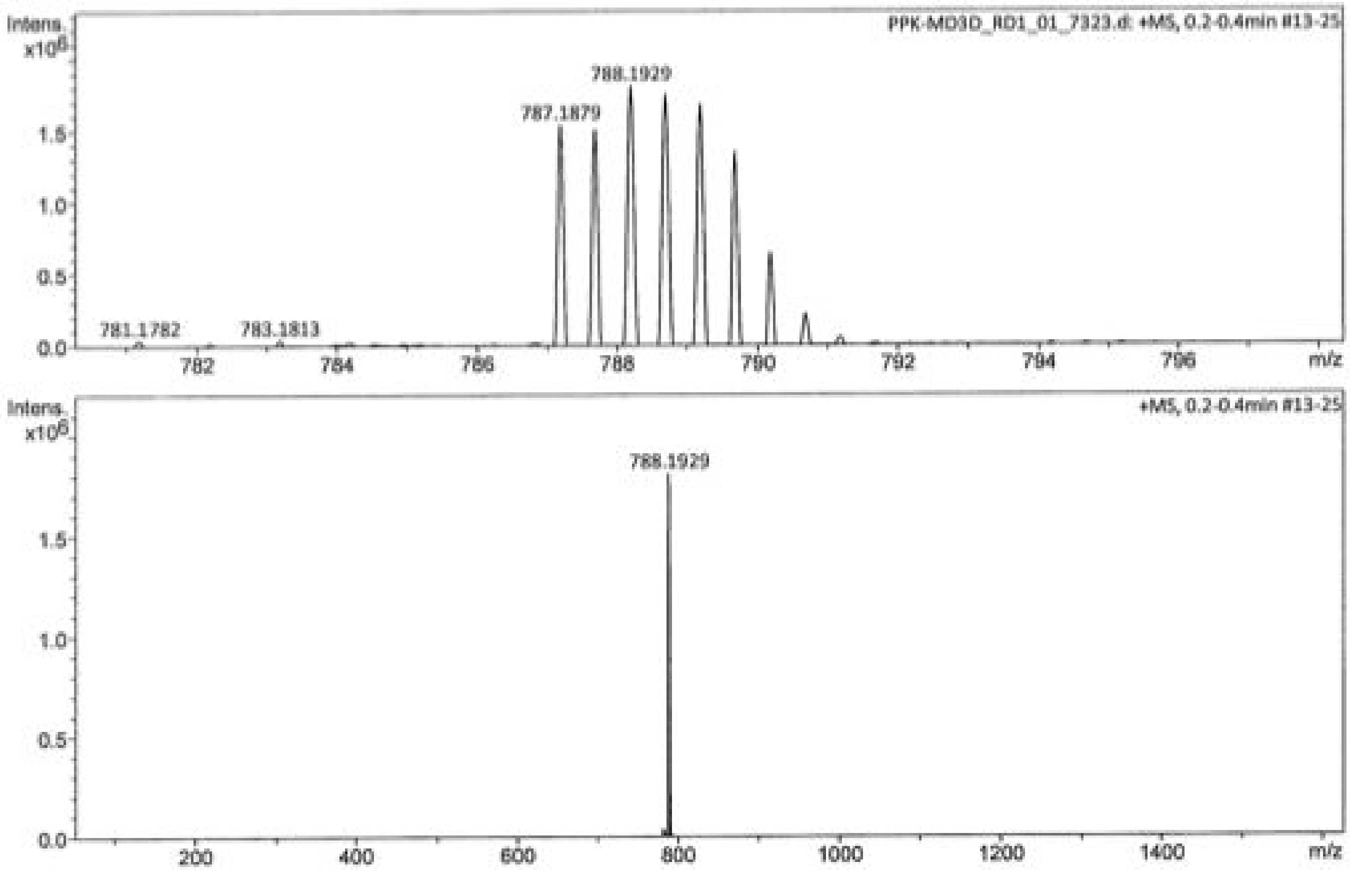

Figure S2. High resolution ESI mass spectra of the homodimer SbP-OCH$_2$O-SbP·(PF$_6$)$_2$.

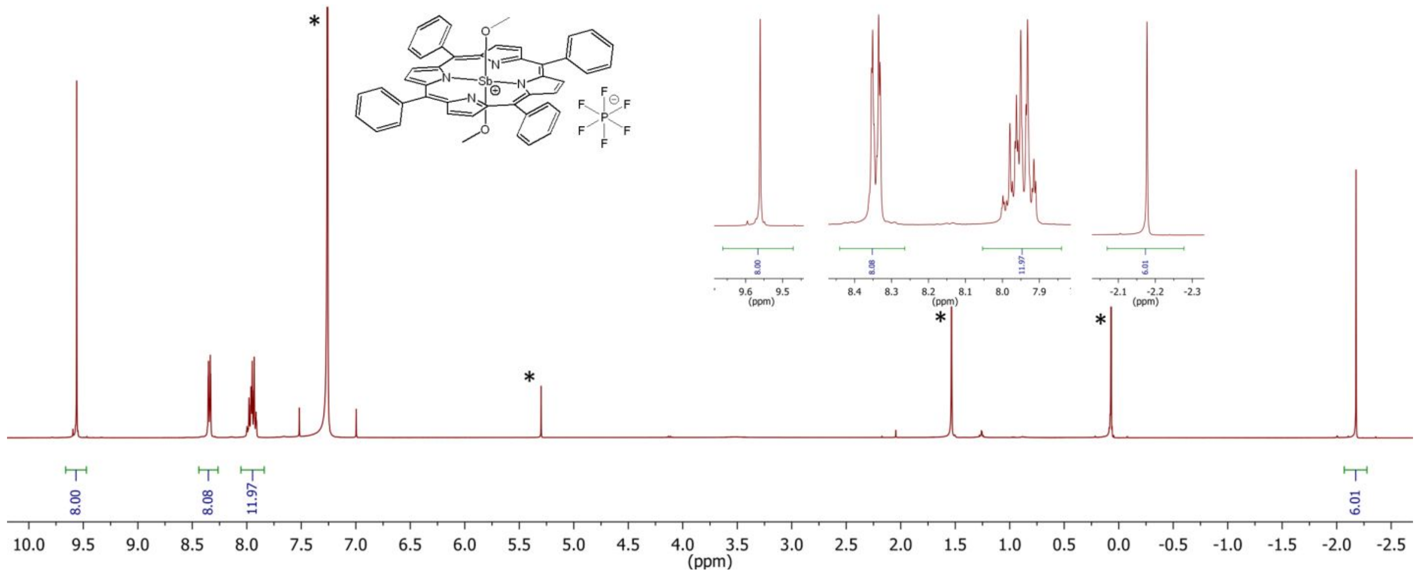

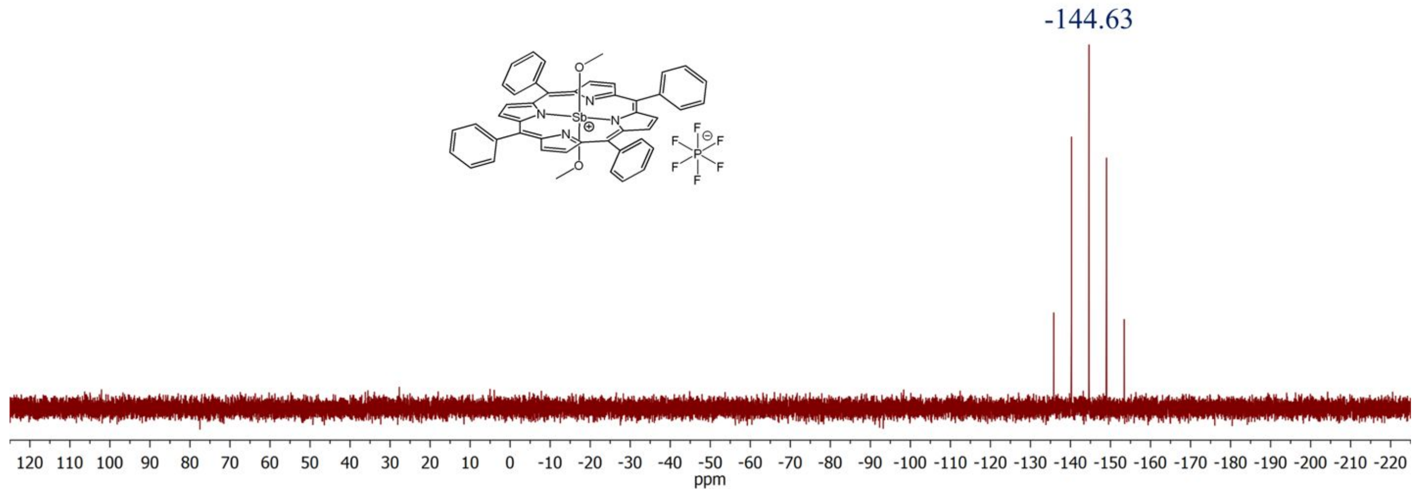

Figure S3. ^{1}H (400 MHz) and ^{31}P (162 MHz) NMR spectra of the $SbP{\cdot}PF_6$ in $CDCl_3$.

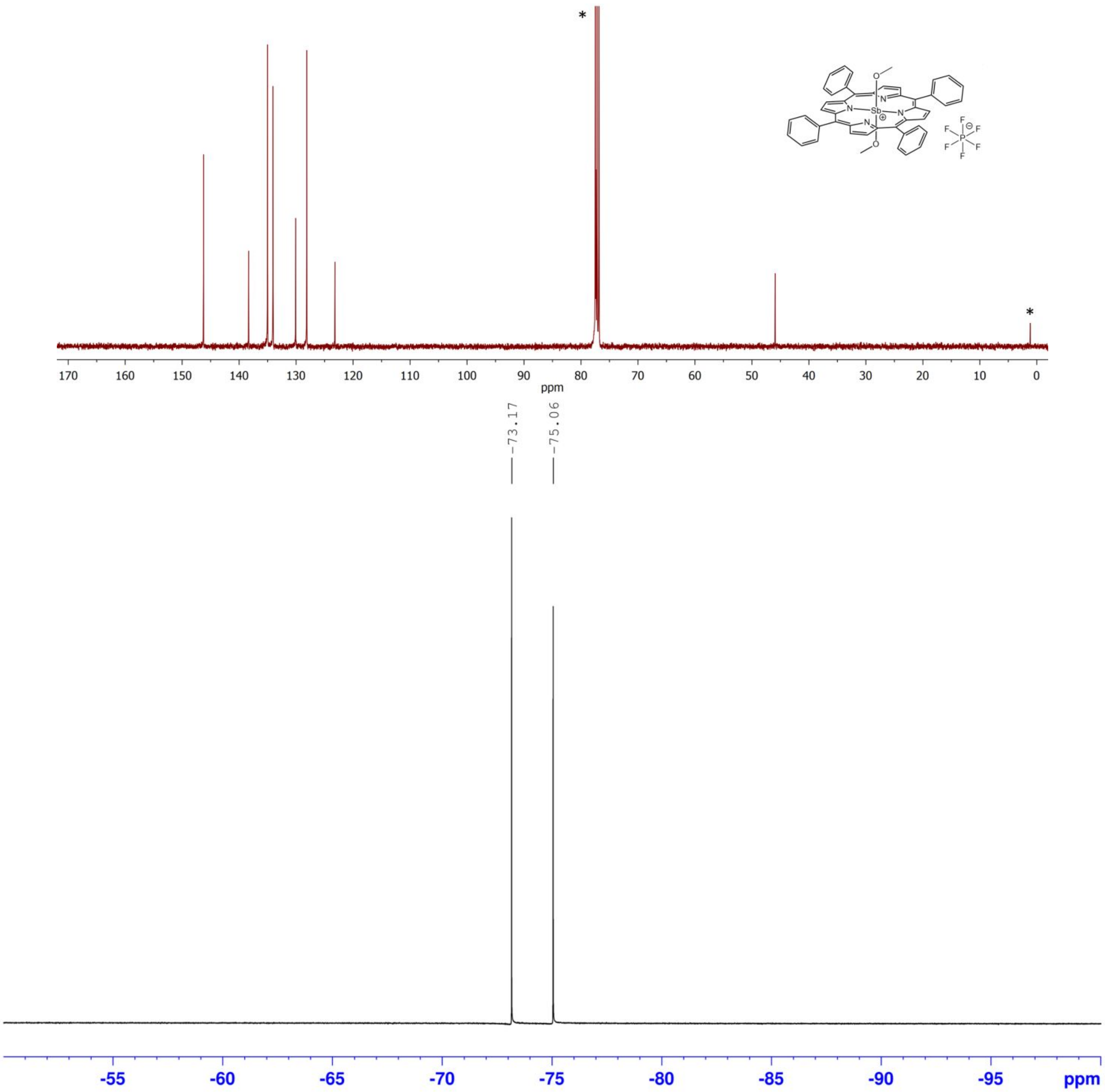

Figure S4. ^{13}C (100 MHz), and ^{19}F (376 MHz) NMR spectra of the SbP·PF_6 in $CDCl_3$.

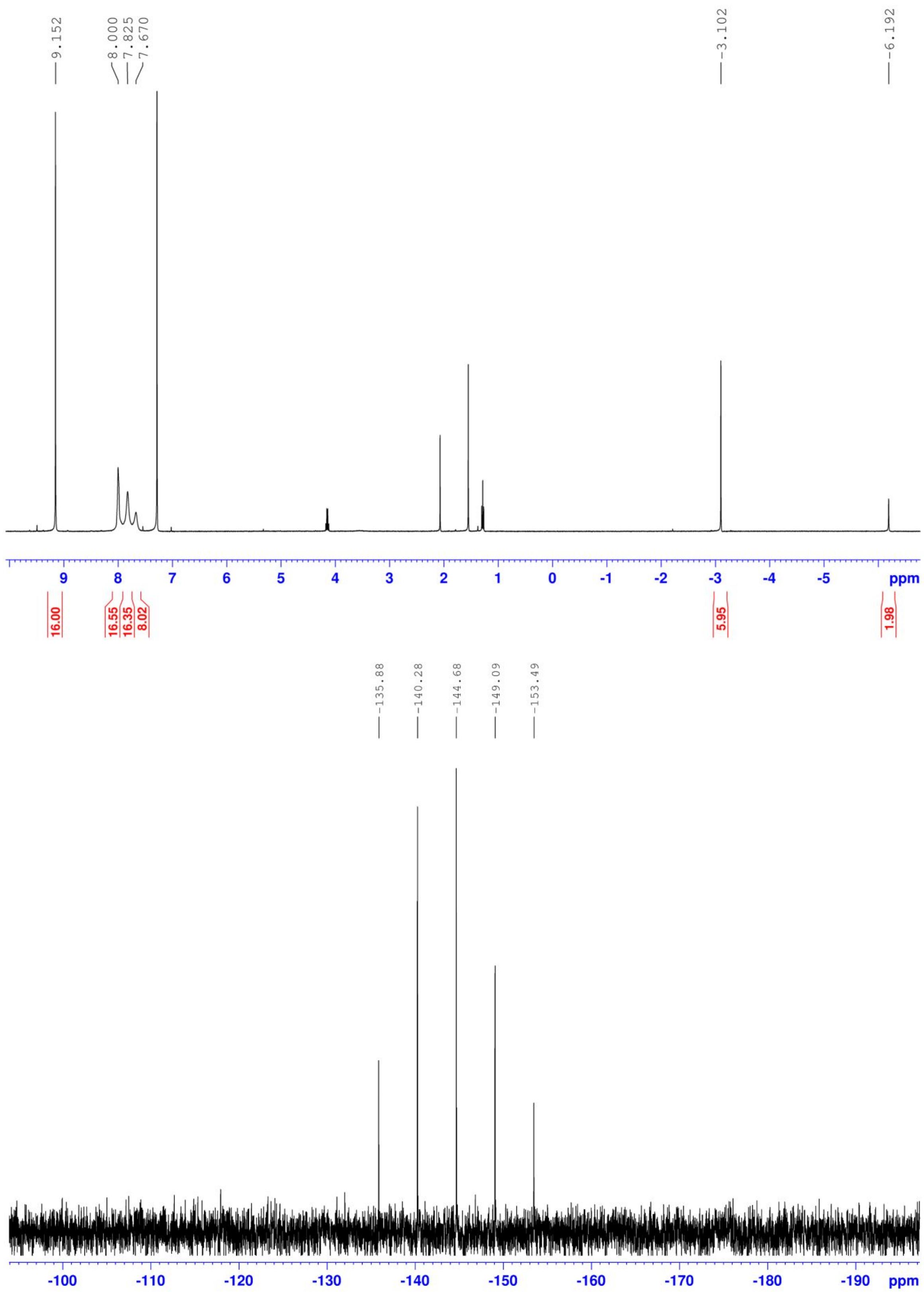

Figure S5. ^{1}H (400 MHz) and ^{31}P (162 MHz) NMR spectra of the SbP-OCH_2O-SbP·$(PF_6)_2$ in $CDCl_3$.

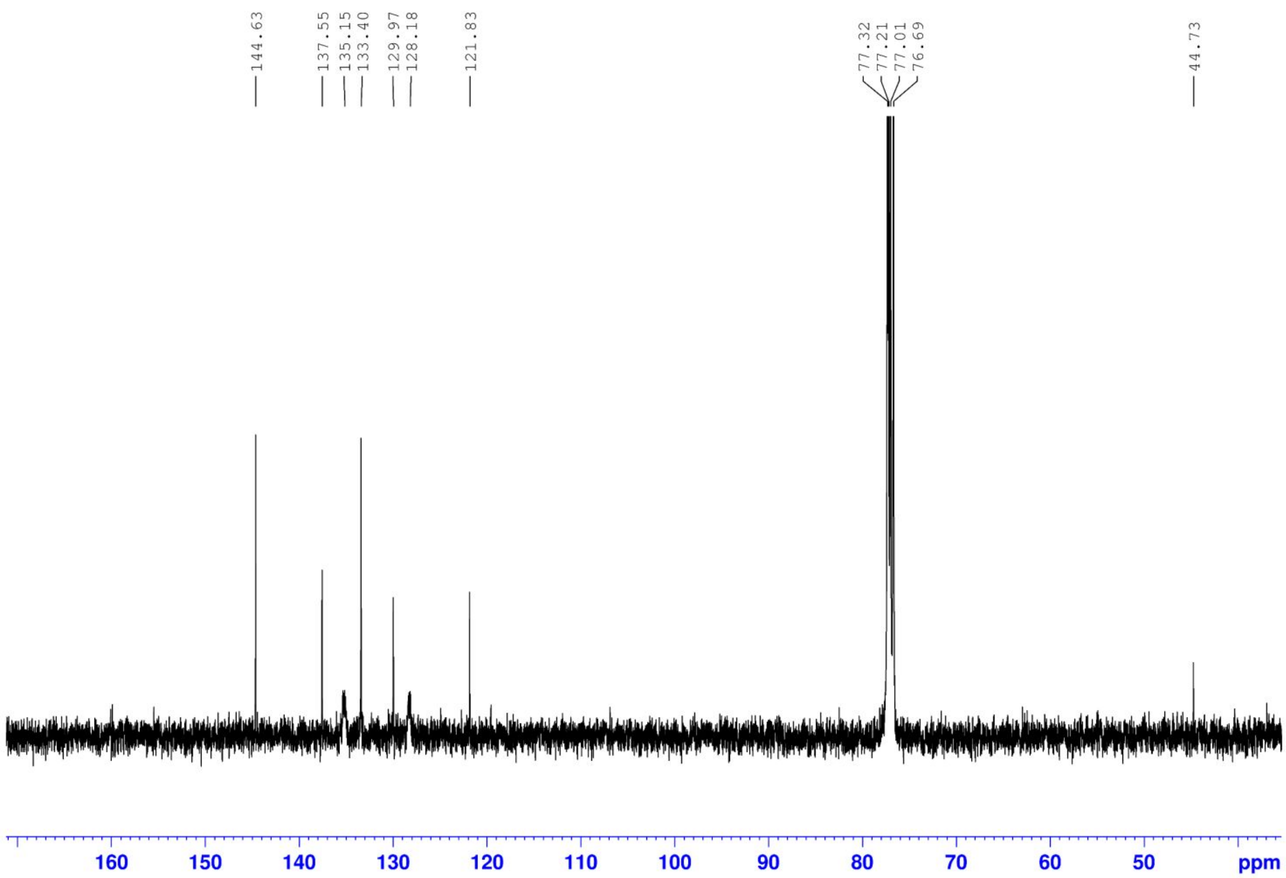

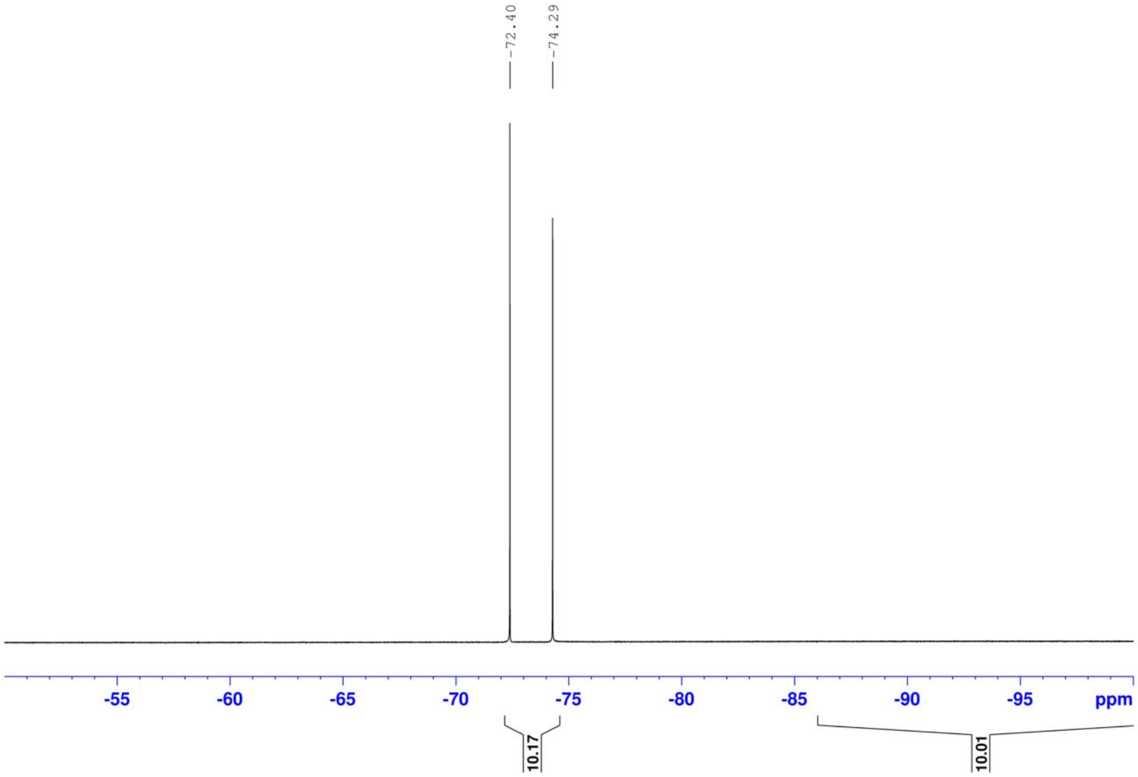

Figure S6. ^{13}C (100 MHz) and ^{19}F (376 MHz) NMR spectra of the SbP-OCH_2O-SbP·$(PF_6)_2$ in $CDCl_3$.

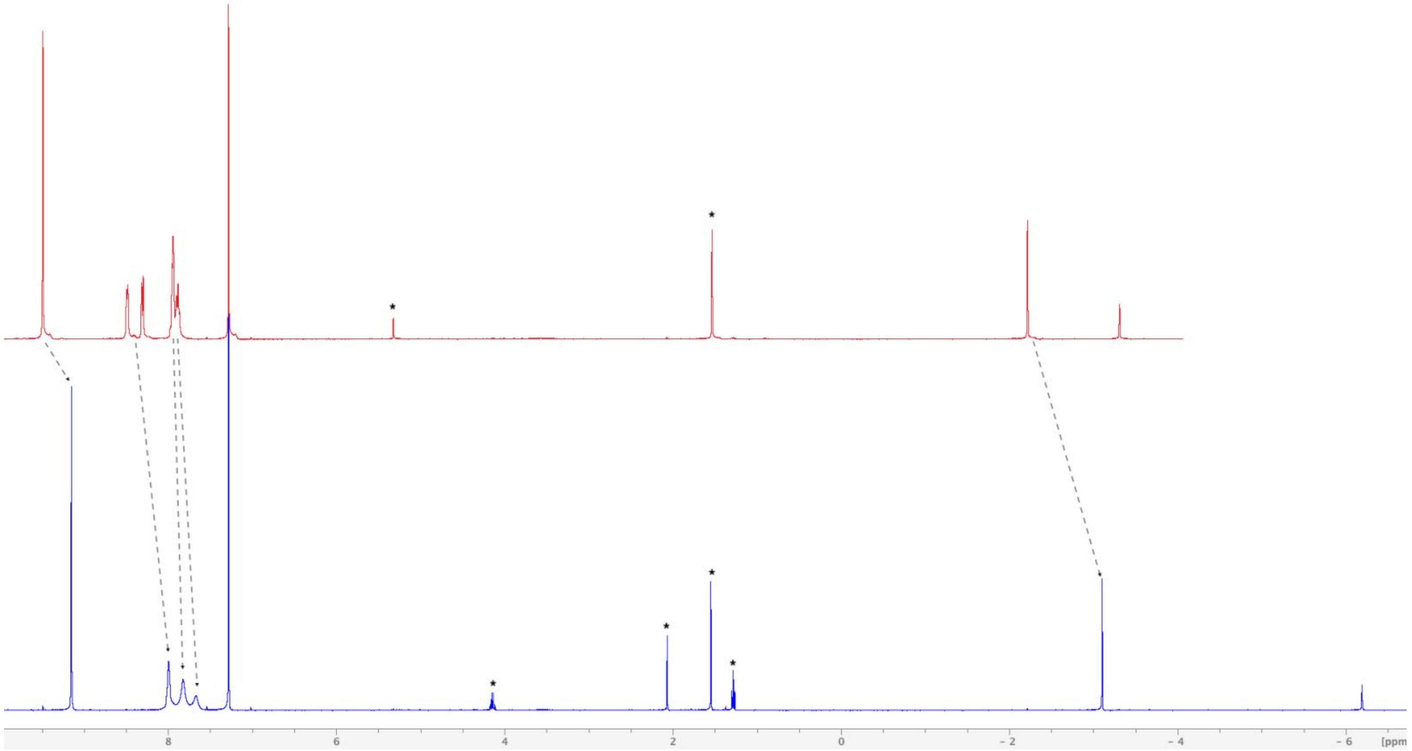

Figure S7. ^{1}H NMR (400 MHz) spectra of SbP-OH·PF_6 (top) and the homodimer SbP-OCH_2O-SbP·$(PF_6)_2$ (bottom) in $CDCl_3$.

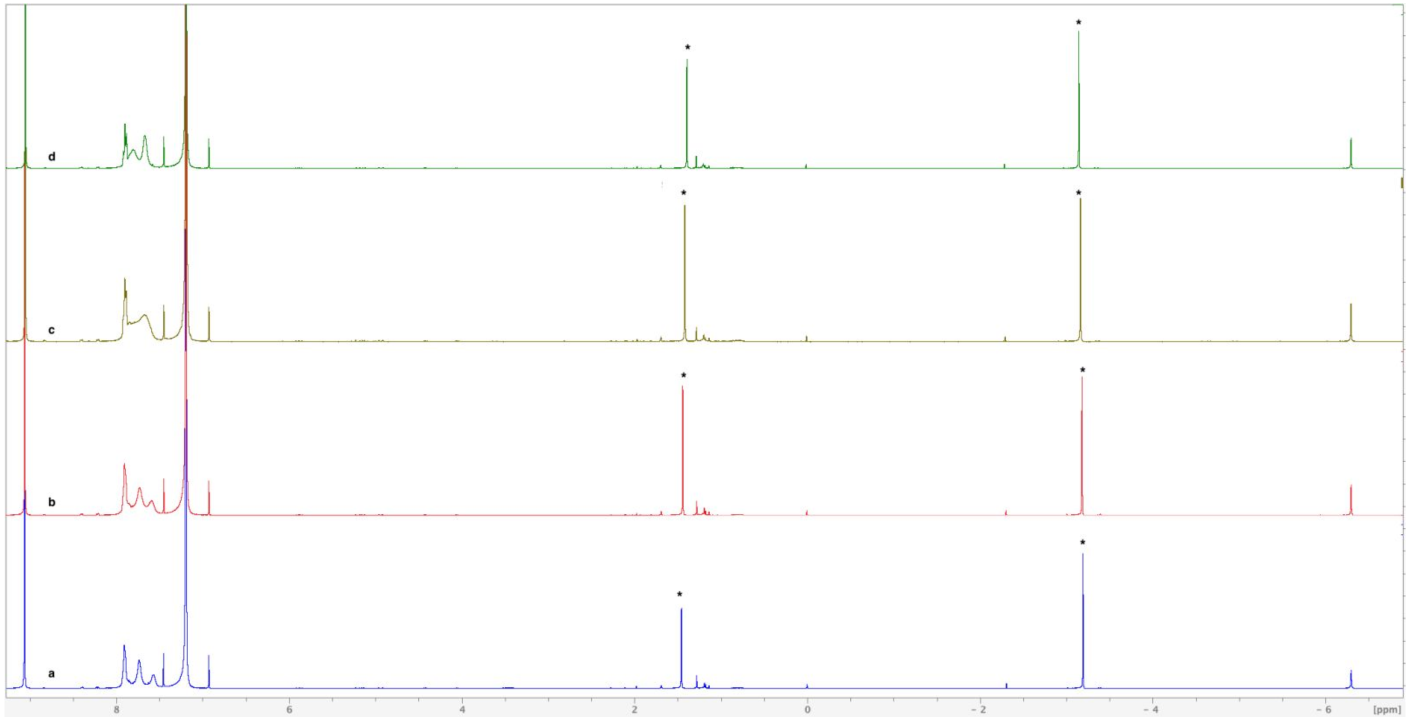

Figure S8. ^{1}H NMR (400 MHz) spectra of the SbP-OCH_2O-SbP·$(PF_6)_2$ at (a) 23°C, (b) 30°C, (c) 40°C, (d) 50°C in $CDCl_3$.

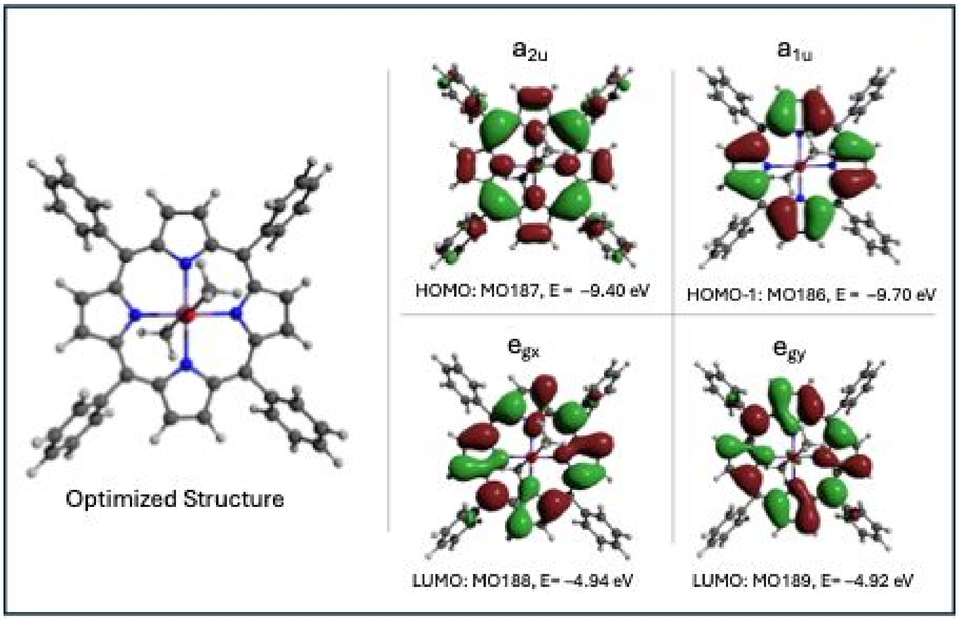

Figure S9. The frontier orbitals of monomer $SbP \cdot PF_6$.

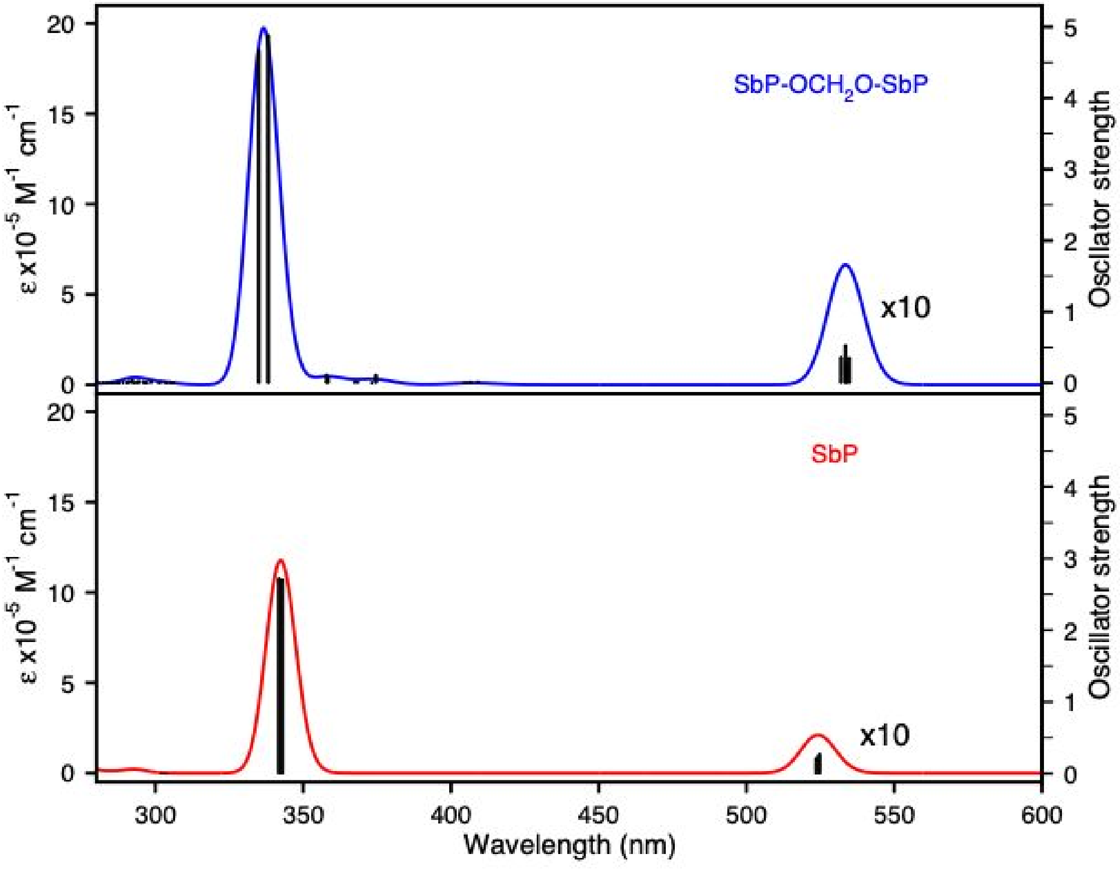

Figure S10. Absorption spectra $SbP\text{-}OCH_2O\text{-}SbP \cdot (PF_6)_2$ and $SbP \cdot PF_6$ from TDDFT calculations. The black bars are the calculated transition wavelengths and oscillator strengths. The red and blue spectra have been obtained by convoluting the line positions with a Gaussian lineshape and converting the intensities to extinction coefficients.

The FWHM of the Gaussian lineshape is 150 cm^{-1} for λ < 450 nm (Soret band) and 100 cm^{-1} for λ > 450 nm (Q-band).

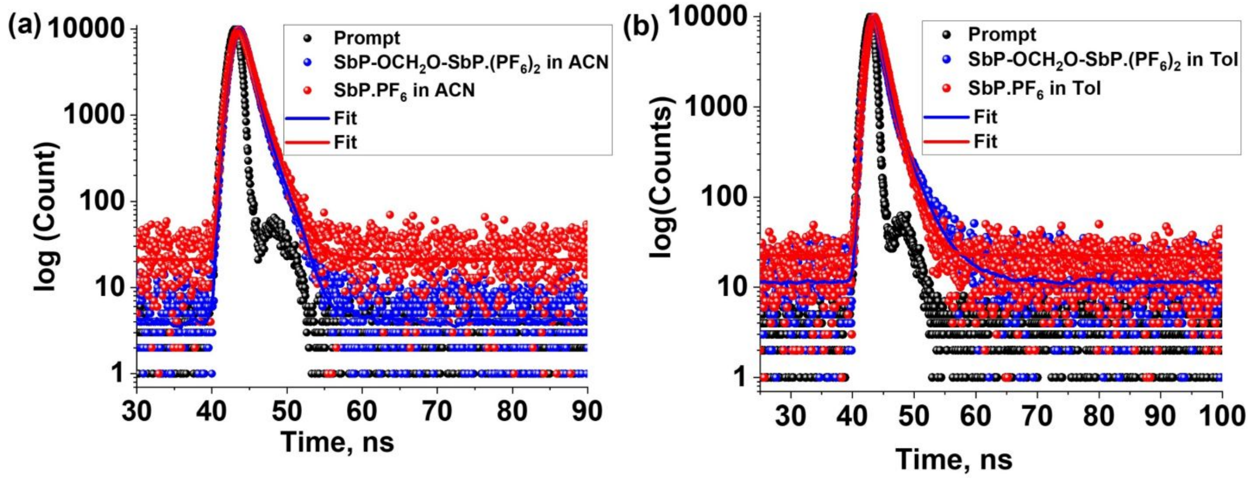

Figure S11. Steady state fluorescence lifetime of 600 nm emission peak with an excitation wavelength of 560 nm.

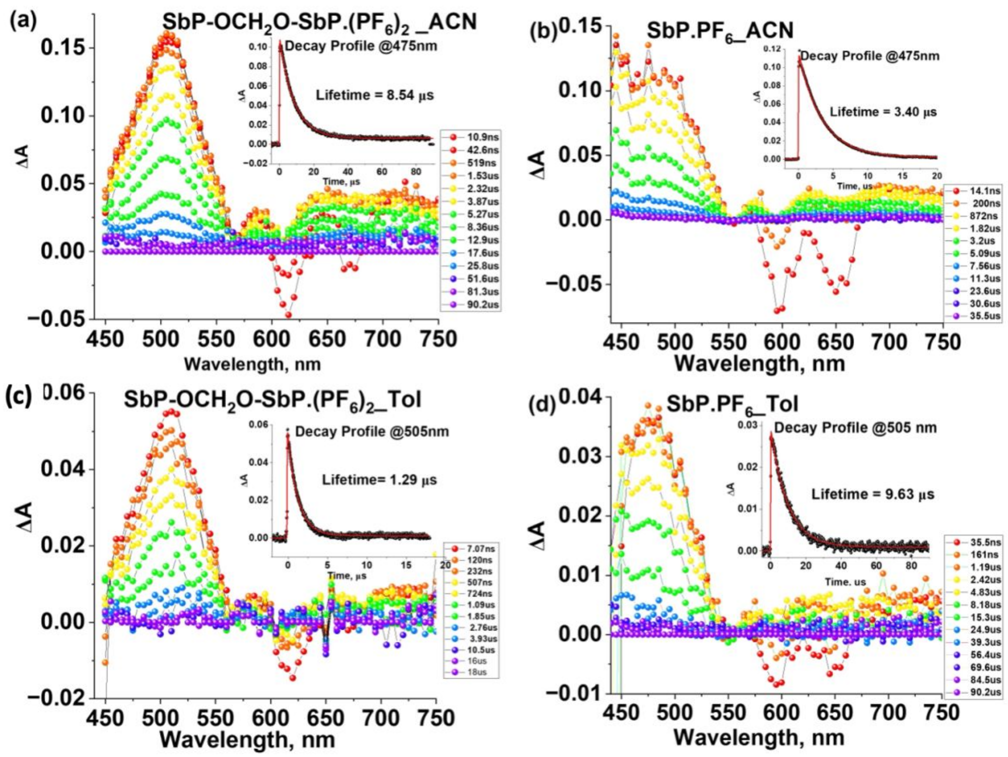

Figure S12. *ns*-TA spectra at the indicated delay time, with the triplet state decay profile in the inset, with the lifetime.

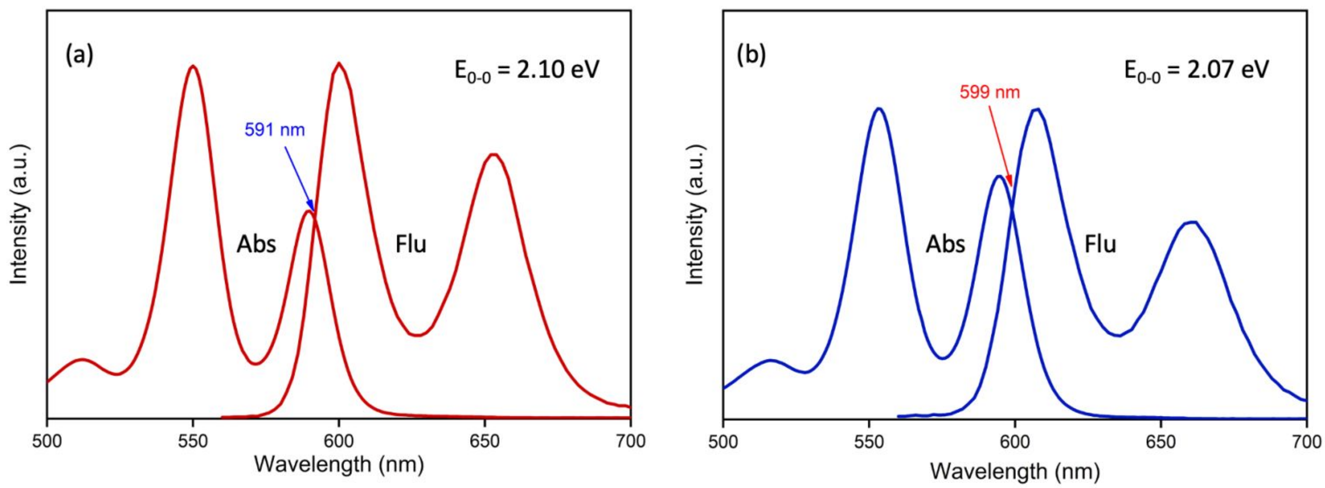

Figure S13. Superposition of the absorption (solid) and fluorescence (dashed) spectra of (a) SbP.PF_6 and (b) SbP-OCH_2O-SbP·$(PF_6)_2$ in CH_3CN.

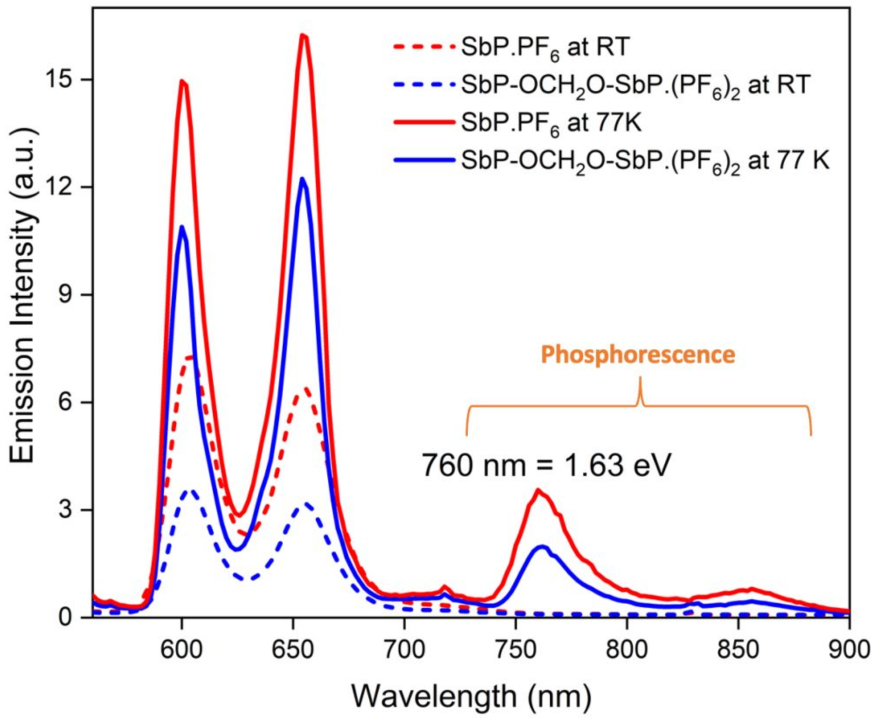

Figure S14. Phosphorescence spectra of monomer and dimer at room temperature and 77 K in THF:1,2-dibromoethane:CH_2Cl_2 (=15:3:2 ratio).

Transient EPR Spectral Simulations

The transient EPR spectra shown in Figure 9 were simulated using EasySpin version 6.0.10. Initial attempts to reproduce the experimental spectra using the EasySpin function "pepper" did not reproduce important aspects of the experimental spectra that arise from the dynamic Jahn-Teller effect that is known to cause broadening of the spectral features from the x and y canonical orientations in metalloporphyrin triplet state spectra.[1] A full treatment of this effect requires a dynamic model involving back-and-forth transitions between two closely spaced triplet

states. A simpler but less rigorous approach is to approximate the effect on the spectrum by including an orientation-dependent linewidth using the DStrain parameter in EasySpin. A second problem is the orientation-dependent net polarization that is apparent in the spectrum. This polarization can be generated by several different possible mechanisms[2,3] but cannot be calculated directly using the function "pepper" in EasySpin. Thus, the spectra of individual orientations were calculated at thermal equilibrium and with selective population of the spin states using EasySpin. The thermal equilibrium spectra were weighted with an orientation-dependent parameter described by vector. The individual orientation spectra were then summed to give the observed powder spectrum.

Table S1. Parameters used to simulate the TREPR spectra of SbP.PF_6 and SbP-OCH_2O-SbP.$(PF_6)_2$.

**SbP.PF_6**	
Principal g-values	[2.00281, 2.00268, 1.99725]
D, E (MHz)	[1019, 331]
DStrain (MHz)	[0, 59]
Gaussian Linewidth (mT)	[0.68, 0.52, 0.41]
ZFS sublevel populations [p_x, p_y, p_z]	[0, 0, 1.0]
Net polarization	[-4.7×10^{-3}, -1.6×10^{-3}, 0.25]

**SbP-OCH_2O-SbP.$(PF_6)_2$**	
g-value	[2.00343, 2.00234, 1.99737]
D, E (MHz)	[1012, 301]
DStrain (MHz)	[0, 59]
Gaussian Linewidth (mT)	[0.19, 0.60, 0.86]
ZFS sublevel populations [p_x, p_y, p_z]	[0, 0, 1]
Net polarization	[-1.8×10^{-4}, -3.3×10^{-3}, 0.22]

169

Coordinates from ORCA geometry optimization of [SbP-OCH2O-SbP]$^{2+}$ singlet ground state

Sb	-2.12030788312651	0.11428922528129	5.23957679464329
O	-1.90319331645817	-0.50329194019500	3.36034305532067
N	-2.09969376942141	-1.87692478027189	5.85606198272860
N	-0.05750634784193	0.20964769230819	5.54315984373032
C	-3.18721901129339	-2.71986672263725	5.82700569774061
C	-2.73086761790504	-4.03199965031539	6.19143150041885
H	-3.36181377179978	-4.91487303605989	6.23770443037604
C	-1.39730872220024	-3.94873555429006	6.46085905254701
H	-0.73102675343363	-4.74993281696792	6.76744427965439
C	-0.99385582856822	-2.58581810957933	6.25645476358603
C	0.30817555747343	-2.08392610982089	6.42303092096659
C	0.71389002340989	-0.77848608616155	6.11718649595455
C	2.03442739474274	-0.24714838810447	6.30018424791210
H	2.85751906709741	-0.78742335464965	6.75883487939348
C	2.04140289543178	1.02688590958312	5.81814777512696
H	2.87214639121286	1.72583017995962	5.81678813231608
C	0.72165093160792	1.32255903638068	5.33289738162040
C	0.31025971334635	2.54610994664793	4.78151913699558
C	1.37911283299919	3.55688955766569	4.54181006688061
C	1.39103657164767	4.77816537127486	5.22431380226451
H	0.60027256123420	5.00420694020684	5.94214999563472
C	2.41818887084111	5.69310566383207	5.01285931403969
H	2.42202657430603	6.63849173601648	5.55853710557889
C	3.44922762965432	5.39459973875470	4.12450249767323
H	4.26035489912751	6.10805252426534	3.96804594882414
C	3.44448978671469	4.18058839608940	3.44220185600372
H	4.24731531856508	3.94294973991721	2.74301520199813
C	2.41253874070643	3.26908574318796	3.64327344510210
H	2.39236866531055	2.32273424359853	3.09860370024511
C	1.34151293570517	-3.02219398059091	6.94866929930562
C	1.26807848195515	-3.48677906693234	8.26577172974751
H	0.45590263935414	-3.14928222540187	8.91300342327590
C	2.23226660529170	-4.36244179009761	8.75563432972837
H	2.17129953548227	-4.71320179489087	9.78742738542160
C	3.27396289877133	-4.78608594364094	7.93408976583749
H	4.02837639823781	-5.47441981821003	8.31937104794418
C	3.35161427737862	-4.32972316729219	6.62047316039050
H	4.16408560367651	-4.66387361107862	5.97256486987786
C	2.39195218706839	-3.44988804002148	6.12986201897333
H	2.44952286408426	-3.09694637222447	5.09773330708116
N	-2.13156663475594	2.10988735902674	4.62644049437073
N	-4.17459920792633	0.02309008223665	4.91980243454410
C	-1.01153086204952	2.89461563844216	4.46340179071128
C	-1.44343816571073	4.16093853642269	3.94455909012120
H	-0.78767590747044	4.98528948749289	3.68617995203150
C	-2.79988266782550	4.12400109640584	3.82551847496772
H	-3.44991998997761	4.91193599590398	3.45913607641197
C	-3.24738436951182	2.83001556423807	4.25251208749152
C	-4.57906952357625	2.39027880287215	4.28075601308092
C	-4.99176820247965	1.09498301086140	4.63942841965830
C	-6.34928721897116	0.65680588562128	4.78344520095625
H	-7.22050812989170	1.29118969818862	4.65207750751744
C	-6.32832387672216	-0.66485215830433	5.11853315461396
H	-7.17787193815356	-1.31187312425856	5.31757764564512
C	-4.95713081330137	-1.07463973273241	5.20265070012262
C	-4.51377012916166	-2.36348047184217	5.54267948134709
C	-5.55493381235879	-3.42727114517032	5.62781690998243

C	-6.23075857128877	-3.83302764164126	4.47143797978738
H	-5.97619126779998	-3.37837053819037	3.51121901943385
C	-7.21024279226744	-4.81861502544499	4.53867379677440
H	-7.72679754277786	-5.13454822209618	3.63041144677277
C	-7.52905096109565	-5.40301787721794	5.76230011633091
H	-8.30014959751214	-6.17370408098687	5.81582481969969
C	-6.86370122847469	-5.00119031814418	6.91786558759978
H	-7.11644859611449	-5.45126444341841	7.87958834540715
C	-5.87896370788182	-4.01990035975445	6.85233431656094
H	-5.36673933450263	-3.69769855936604	7.76127074539863
C	-5.64121590297309	3.36381174314863	3.90000183162678
C	-6.47457390401376	3.10370651655344	2.80503618655650
H	-6.34823460463433	2.18187061240904	2.23742000447685
C	-7.46181079977669	4.01276898207584	2.43937498199981
H	-8.10089897857555	3.79769697016024	1.58095778606068
C	-7.63509421991215	5.18800659821329	3.16672707399887
H	-8.41276251938727	5.89920400981991	2.88243789530054
C	-6.81849089331962	5.44904387634995	4.26436460580919
H	-6.95963269422015	6.36115908670213	4.84702951397885
C	-5.82639092260260	4.54382889292108	4.62888421261282
H	-5.19782855570305	4.74602908372815	5.49846964130911
O	-2.38901318442082	0.56545028625484	7.12901941644246
C	-3.00653660244224	1.74140430088759	7.58886502090936
H	-4.05759343725723	1.82321665904211	7.25851210422798
H	-2.47121091972126	2.65466408308573	7.27568148944391
H	4.35271753744515	-2.43803736090578	0.28120822782304
C	3.28947901737265	-2.25989382181859	0.14839392598334
C	2.66776246590634	-0.96653599817737	0.17860558260800
C	3.32908481774848	0.26372426574647	0.30362109699903
C	4.81477694825185	0.25376059595614	0.41849806235778
C	5.42330528132802	0.70932504560371	1.59378496628461
H	4.80163191442495	1.04982689145344	2.42498337653530
C	6.80933300067773	0.71468429119816	1.71180467755680
H	7.27488085855637	1.06401874782579	2.63529722249330
C	7.60137471479734	0.27263881188425	0.65457039642922
H	8.68904096841562	0.28050800334027	0.74548032789341
C	7.00337670257915	-0.17761346039648	-0.51981366756328
H	7.62107160447723	-0.51672901362879	-1.35334647765963
C	5.61676957764174	-0.19010869513422	-0.63776792179626
H	5.15060068423955	-0.53219400178080	-1.56410148615130
C	2.70495369817249	1.52286952474291	0.30046284154710
C	3.37737787412626	2.78204830076942	0.17519507297262
H	4.45269473944455	2.89850417832554	0.07400852409481
C	2.42957470374749	3.76338416235294	0.17166067024150
H	2.58782954813928	4.83314137170743	0.07517409725426
C	1.14800193137613	3.13732247612058	0.31338098886209
C	-0.08848056438752	3.80198712954945	0.39751508805892
C	-0.05576255532279	5.29138754048726	0.45039716184162
C	0.59846569078197	5.94182524413178	1.50437460735561
H	1.09283513977894	5.35391843512359	2.27837015560623
C	0.62358145013061	7.33109693846918	1.56780820510175
H	1.13417311440936	7.82369199816464	2.39745956992609
C	0.00386376194176	8.08763044588411	0.57587456340530
H	0.02734357018917	9.17787934680356	0.62294869548131
C	-0.63836607667719	7.44874908000253	-0.48201718641196
H	-1.11261411222002	8.03695636906771	-1.26975300558330
C	-0.67010735827086	6.05877139792595	-0.54500152988143
H	-1.16211111594593	5.56213179923688	-1.38371574333039
C	-1.34215757408781	3.17399833301949	0.45554487251198

C	-2.60627588882591	3.83717600950805	0.61216306540448
H	-2.73147587863561	4.91059551509532	0.71242362299493
C	-3.58051931533241	2.88623713698659	0.63063855430238
H	-4.64649600631897	3.04493146996123	0.75331814985240
C	-2.94913742573132	1.60653138434437	0.46525530294693
C	-3.60688149762909	0.37234957772497	0.34799971860576
C	-2.98443299604908	-0.85600078155871	0.07678900949324
N	-1.63344500724701	-1.10053169328807	0.02162028069159
C	-1.42693087112536	-2.42586206043418	-0.30003614790412
C	-0.19444669423413	-3.08875338184715	-0.36816219091855
C	1.06232626653677	-2.48951752102401	-0.17239311230296
C	-0.21326975875524	-4.55623619604666	-0.63503943891047
C	-0.73485297904524	-5.44385304494943	0.31196353874854
H	-1.12643146384237	-5.05705116656196	1.25547053866100
C	-0.74406141954410	-6.81247620067789	0.06183442798220
H	-1.14671738675588	-7.49859844803898	0.80923262044782
C	-0.23741287443262	-7.30653459430553	-1.13782966671116
H	-0.24738774105662	-8.38006194096650	-1.33488432084850
C	0.28197000106129	-6.42857473509281	-2.08590362444752
H	0.67499272078819	-6.81136210521228	-3.02951005294443
C	0.29754644115172	-5.05984691479702	-1.83556462381040
H	0.69887547426840	-4.37179463844250	-2.58254117264821
C	-2.71468527197524	-3.03195926386684	-0.48502232303549
H	-2.87298108958162	-4.06660359608491	-0.77512547425588
C	-3.66097416998864	-2.08166635021677	-0.24611591067815
H	-4.73880037223960	-2.19455599738537	-0.31170884226111
C	-5.09125365564677	0.34337232831468	0.47697132847530
C	-5.67743451728401	-0.40084320748057	1.50730622965434
H	-5.03274329030978	-0.90877738761812	2.22803336955879
C	-7.06243066996354	-0.48393599881222	1.61425776943927
H	-7.50864259651019	-1.06955466437359	2.41918705654911
C	-7.87654038831726	0.18435226814244	0.70312638384625
H	-8.96275611222451	0.11636708437180	0.78613627951540
C	-7.30000398828252	0.93489894371312	-0.31946547106664
H	-7.93283738976029	1.45167696565973	-1.04335231059629
C	-5.91511895745113	1.01106600165092	-0.43553128939157
H	-5.46750960262521	1.57531927539350	-1.25584354727019
N	-1.59400517993497	1.82163030703445	0.38522673421983
N	1.35271829705557	1.77635257283767	0.36694476663742
N	1.31440836793718	-1.14858281565877	-0.00301941772685
Sb	-0.14420069872519	0.34443519976359	0.19165763695253
O	-0.02543928771814	0.12543621341710	2.16519287912479
C	-0.67279299436577	-0.90470237007875	2.84417202774394
O	-0.25286072092193	0.59068421995941	-1.75113988819859
C	0.87016667925319	0.67734422326034	-2.59290247741304
H	1.47563097815534	1.58002674447632	-2.39705012236011
H	1.52712617101772	-0.20686135486841	-2.51576742140025
H	0.50473280503827	0.73577392183922	-3.62833253667958
C	2.31380308733630	-3.18588229008894	-0.07730950680613
H	2.42826729203305	-4.26286425790398	-0.16136330019797
H	-3.00313549779875	1.70847163743240	8.68799858170585
H	0.01478250722959	-1.24319607374644	3.63472945797319
H	-0.86981003373013	-1.76881476854270	2.19074188545346

169

Coordinates from ORCA geometry optimization of [SbP-OCH2O-SbP]$^{2+}$ triplet state

Sb	-2.15385404684413	0.11237586716583	5.24018962402566
O	-1.82943676549471	-0.50828535029286	3.37939261653413
N	-2.13479905447101	-1.89818793586664	5.85619724459187
N	-0.10266470763107	0.24136268574767	5.63172920643286
C	-3.17258094100642	-2.79091480284473	5.68591829435176
C	-2.67686743385265	-4.10768931787165	6.00318829703941
H	-3.25560054736476	-5.02322698855650	5.92627905301764
C	-1.38629417375505	-3.97859696157725	6.40678989013360
H	-0.70937752333658	-4.76806058993774	6.71989737650987
C	-1.03290772355260	-2.58270959488936	6.31971296448403
C	0.22441273516416	-2.04468079215167	6.60902833191380
C	0.64526269399737	-0.69906393847209	6.31790130642409
C	1.90556773751053	-0.14739963329475	6.58334646043073
H	2.70600989644813	-0.63761744911415	7.12865985504784
C	1.93195173022276	1.12928544044358	6.02001449253349
H	2.75358338582030	1.83848889153520	6.04541293215390
C	0.68200036876554	1.35318085141476	5.42998260683364
C	0.27030733738506	2.57103062024695	4.76800001188993
C	1.35720624495924	3.53820311921973	4.47883312031425
C	1.38673516736080	4.80148969565856	5.08122573245383
H	0.59302577749303	5.08965449065002	5.77280261653625
C	2.43999606137228	5.67594522762899	4.83001756880976
H	2.46099202532438	6.65230360273384	5.31766228680281
C	3.47244437013153	5.29868946391333	3.97461583079269
H	4.30198171331412	5.98213225382436	3.78423731294902
C	3.44768210185320	4.04363765904229	3.36933183261569
H	4.25284285259826	3.74686629054712	2.69597625408770
C	2.39853048170927	3.16582145222141	3.61854739391543
H	2.36638553379867	2.18440151931706	3.14024578783452
C	1.22592232174499	-2.94453563647157	7.23037979708891
C	0.97021079026257	-3.52599722069046	8.47898051097357
H	0.03938427319774	-3.29596848044900	9.00110412440005
C	1.90576517729610	-4.37180332936653	9.06519610835716
H	1.70288315454543	-4.80705485465652	10.04530565460312
C	3.09808031308512	-4.65925555042523	8.40551788162082
H	3.82910789546616	-5.32738723386678	8.86448822627067
C	3.35662730048487	-4.09260891614765	7.15894264814085
H	4.28634070087537	-4.32353750262476	6.63578377246124
C	2.43188870823103	-3.23337275290353	6.57728468530799
H	2.63386195906653	-2.79521031164580	5.59822329802229
N	-2.15790460716777	2.12844795360018	4.64520631136893
N	-4.18154013042984	-0.01697838041197	4.82866101712105
C	-1.03934296697021	2.91344042342241	4.44498178209022
C	-1.48057855439566	4.17334897465863	3.90405872381821
H	-0.82879636082193	4.98902878660306	3.61007790117809
C	-2.83650570403561	4.14055291698907	3.82725126904561
H	-3.49291280255594	4.92272371372282	3.46010346111639
C	-3.27807960013941	2.85083160034548	4.28990205418763
C	-4.60289723984168	2.40506272117889	4.33285502670479
C	-5.01956187175489	1.07179721653496	4.66439530382178
C	-6.34145518071542	0.63835539782925	4.83273851930824
H	-7.22313301576570	1.26862027152385	4.77157324816695
C	-6.30514394982851	-0.73343454719907	5.07892740903976
H	-7.14916013859318	-1.39186126386958	5.26196560378946
C	-4.96012449758742	-1.12330320721106	5.07227376534507
C	-4.47781757980162	-2.46554848206343	5.32382164749753
C	-5.48884195503657	-3.54373498490788	5.23173356745829

C	-6.17754708954306	-3.74870154015680	4.02719092363161
H	-5.95029033676348	-3.12103005452698	3.16345359889180
C	-7.12699704407465	-4.75762711841385	3.92307832810185
H	-7.64590827639606	-4.91942451784710	2.97648096114885
C	-7.41841508991750	-5.55981451713800	5.02528039775084
H	-8.17198260607912	-6.34549727679090	4.94567798881131
C	-6.75218040623146	-5.35271492335326	6.23030832779117
H	-6.98933246589053	-5.96801159728850	7.10000948235530
C	-5.78833632078782	-4.35477520494345	6.33367964452546
H	-5.28294711372522	-4.18154380317701	7.28552071220571
C	-5.66873249513779	3.37285121472532	3.98109839581385
C	-6.54835550961044	3.09732918598251	2.92376192417627
H	-6.45358505184300	2.16249860541713	2.37206591646087
C	-7.52962474593660	4.01555151510664	2.56939930731745
H	-8.19883591434196	3.79347502092585	1.73618610613874
C	-7.66147849976440	5.20860223635791	3.27742358885625
H	-8.43940331069951	5.92414366413075	3.00480114570244
C	-6.80399787671125	5.48250388505077	4.34050309034521
H	-6.91467163720549	6.40759312118890	4.90902089192710
C	-5.80869792083239	4.57495251065829	4.68705469327826
H	-5.14694030133903	4.78685702785608	5.52888393797138
O	-2.61515597672453	0.60765704254637	7.08055487811054
C	-1.92286680066832	1.58622408652430	7.81650293303707
H	-1.90880007689249	2.56696997375278	7.30849659491096
H	-0.88186635934983	1.29173475239228	8.03555770210373
H	4.32820517430317	-2.40004899542446	0.20084958908192
C	3.25916746201085	-2.23144315084854	0.10753827913826
C	2.63127821219669	-0.94087486923363	0.12877013708795
C	3.29181012902622	0.29401363928560	0.20445133838186
C	4.78125747290308	0.28726215325211	0.26704590556980
C	5.43086777925849	0.73502211719891	1.42298583908069
H	4.83900178887826	1.06752660893465	2.27887910720506
C	6.82037779356738	0.74143004931946	1.49127806118175
H	7.31823405156873	1.08449603865471	2.40016171202866
C	7.57443157984753	0.30788153447410	0.40320338941430
H	8.66465503708426	0.31648768054774	0.45509343098453
C	6.93508984583309	-0.13563979301232	-0.75180788924298
H	7.52273268766819	-0.46924495892378	-1.60898139311746
C	5.54504957042726	-0.14919264901802	-0.81999217193048
H	5.04614541684368	-0.48771689999893	-1.73051438377095
C	2.66467533094000	1.55106848501794	0.20472268394073
C	3.33143049924162	2.81206109015460	0.06282424182662
H	4.40392754433074	2.93100305592644	-0.06282216772715
C	2.38206553082089	3.79119135303943	0.08313350954666
H	2.53612179427010	4.86135833717048	-0.01583755824937
C	1.10481371761864	3.16161080201975	0.25359905860330
C	-0.13094935050822	3.82332367146736	0.36440957098998
C	-0.09723516260590	5.31283390042427	0.41579931779079
C	0.57504098226819	5.96006893925878	1.46029402897225
H	1.08037001533048	5.36918550200315	2.22524486746959
C	0.60408401842684	7.34917612875063	1.52593891676253
H	1.12869060372731	7.83956493396859	2.34814531964229
C	-0.02920893926381	8.10869181258414	0.54487155841296
H	-0.00245774754861	9.19881766296100	0.59320552603444
C	-0.68897717473439	7.47289626673126	-0.50407635838211
H	-1.17376284663121	8.06343911206899	-1.28362709470865
C	-0.72510459342053	6.08300710645410	-0.56879596971574
H	-1.23110423938445	5.58898968031889	-1.40073404840180
C	-1.38279164940327	3.19409803013602	0.45138261523673

C	-2.64570150726532	3.85821530823571	0.61522199657400
H	-2.77024436103205	4.93272875917307	0.70457094953857
C	-3.61925371584823	2.90698769649963	0.65402830394953
H	-4.68477256927153	3.06439185292199	0.78671377373011
C	-2.98830702835099	1.62551005898462	0.49970744114860
C	-3.64811712846286	0.38996686983089	0.41074358631740
C	-3.02526629200447	-0.84701721409533	0.18246329961059
N	-1.67382034716373	-1.09269818894825	0.12482203973258
C	-1.47038627584357	-2.42777765810210	-0.15717576185054
C	-0.23819005326585	-3.09018015604697	-0.24091374011202
C	1.02210506290369	-2.48076044153242	-0.11341220472983
C	-0.26227689455612	-4.56643602772058	-0.45408346111871
C	-0.75439555055109	-5.41864580381991	0.54023155553561
H	-1.11831903944388	-4.99721335526857	1.47999346535848
C	-0.76981915641615	-6.79557372703779	0.34117682268262
H	-1.14913189887706	-7.45354158518536	1.12523723901312
C	-0.29909226041300	-7.33375261794192	-0.85415449254603
H	-0.31390249227042	-8.41383185423258	-1.01108011880173
C	0.19046352519253	-6.49137454353295	-1.84922058720515
H	0.55504941857840	-6.90867236870747	-2.78961170775426
C	0.21233016068411	-5.11429869639899	-1.65014926421395
H	0.59002209912156	-4.45456618709141	-2.43411143586788
C	-2.75917845009664	-3.04179275909835	-0.30295215233590
H	-2.91939458433068	-4.08619672675851	-0.55442365537107
C	-3.70363729831524	-2.08356576669800	-0.09104444553987
H	-4.78177718333059	-2.19882127906725	-0.14478417863844
C	-5.13524414723909	0.37371189007871	0.51296406694902
C	-5.75250308822301	-0.34205413128780	1.54469595904417
H	-5.13194500959533	-0.83576211587406	2.29490298328947
C	-7.14029519584300	-0.40986014757574	1.62240934967297
H	-7.60880202221104	-0.96936184310455	2.43328600590481
C	-7.92709740523013	0.24489374362429	0.67802040488223
H	-9.01557979642284	0.18930454807739	0.73806691977528
C	-7.31999856877241	0.96784565125925	-0.34696187502509
H	-7.93085977758577	1.47479298709678	-1.09623532734360
C	-5.93221354832505	1.02927729203103	-0.43224701737960
H	-5.46076214256487	1.57255189865047	-1.25342082579035
N	-1.63369295210258	1.84044396099289	0.40304052903261
N	1.31370481519030	1.80173971200204	0.29962625083348
N	1.27276488978413	-1.13382412896048	0.00635218982672
Sb	-0.18415026580049	0.36219113899613	0.20354056754026
O	0.00795736102930	0.20863109224547	2.17750682034992
C	-0.56579273866706	-0.83816895993099	2.89454589026868
O	-0.35337349271839	0.55345659626154	-1.74095217822821
C	0.74341801580905	0.62757754658248	-2.61813543139805
H	1.34813740788491	1.53822083236473	-2.46124590633851
H	1.40819495050274	-0.25071738620901	-2.54054399746928
H	0.34630891052118	0.65966554606181	-3.64300120264461
C	2.28058258018469	-3.16822746837316	-0.04983843133036
H	2.39793297505431	-4.24669958038578	-0.10615900701218
H	-2.44962018122302	1.71172149957199	8.77348764206647
H	0.13614186338654	-1.08527485691791	3.70564412393110
H	-0.68828446135201	-1.74214966822169	2.27650816921748

87

Coordinates from ORCA geometry optimization of SbP^+

Sb	0.00000511462689	0.00000328046765	0.00000049975807
O	-0.07147635936402	0.01284755397163	-1.96317870697147
N	-0.70788200413132	-1.96305580477727	0.07933399808358
N	1.95625260092554	-0.72664312856413	0.02838100193291
C	-2.03586063848905	-2.32162983918977	0.08139105639659
C	-2.10038590996572	-3.75299851569663	0.18699845585557
H	-3.02158373268160	-4.32748657899063	0.21787580017359
C	-0.82324659421758	-4.22456297323841	0.24247527793278
H	-0.50058617485610	-5.25896131979864	0.31732486045503
C	0.06355609496167	-3.09587720012951	0.18695078794102
C	1.46430964194439	-3.15229519166036	0.23007127076905
C	2.32260087403642	-2.04439965807939	0.17396155550816
C	3.75300850681741	-2.08985511909321	0.29121785461666
H	4.33104782126196	-2.99944400244013	0.42660109029019
C	4.21705037912614	-0.81045456637291	0.22378867787581
H	5.24729975110179	-0.47399091213176	0.29456584628678
C	3.08532541774921	0.05932843741294	0.06411025151946
C	3.14052308933761	1.45977828099636	-0.00562457738938
C	4.49538150995156	2.08804255819483	0.00933554954476
C	4.93800964771069	2.78850628782823	1.13539287745897
H	4.28901117186826	2.86900184049103	2.01001597144745
C	6.20178738505773	3.37109439947552	1.14783094269966
H	6.54075020387800	3.91114818829426	2.03385512251835
C	7.03249234681001	3.26195111943170	0.03544818892448
H	8.02305521822689	3.72044555588604	0.04561695256505
C	6.59721361153984	2.56654728853731	-1.08982998979703
H	7.24335309261886	2.48192703064160	-1.96559354219665
C	5.33510961886033	1.98041559626476	-1.10320801106643
H	4.99156816473430	1.43966851135643	-1.98762642087965
C	2.09382323885578	-4.49958058432262	0.36148247901649
C	2.00847444412565	-5.20530545573744	1.56534792589210
H	1.48391354664863	-4.76150883325420	2.41415253271103
C	2.59781154789584	-6.46019468737574	1.68623329587050
H	2.53087850310342	-7.00120722258847	2.63196961095541
C	3.27452209137673	-7.02163068527755	0.60625768193924
H	3.73557128446684	-8.00641758169414	0.70187443718106
C	3.36290180618613	-6.32392651670836	-0.59568221722118
H	3.88947164223824	-6.76156672843554	-1.44586275652900
C	2.77682089082790	-5.06764592710636	-0.71803458346563
H	2.84201386562133	-4.52242413935599	-1.66209215917344
C	0.48045581807215	-0.99874646345663	-2.76530731640760
H	0.03860092978028	-1.99071912416067	-2.56140187831291
H	1.57638417696876	-1.07833058039395	-2.65327961856825
H	0.26612991427353	-0.74584200046596	-3.81415335844459
N	0.70788873793650	1.96306290328220	-0.07933473853000
N	-1.95624427425791	0.72664734334253	-0.02837742844945
C	2.03586771683964	2.32163577627493	-0.08139744045797
C	2.10039295934090	3.75300469857111	-0.18700438665082
H	3.02159087788490	4.32749221793642	-0.21788622010594
C	0.82325397422719	4.22456896955713	-0.24249179197314
H	0.50059425121655	5.25896685243309	-0.31735144171294
C	-0.06354905632945	3.09588422828128	-0.18694895105812
C	-1.46430313668326	3.15230141081453	-0.23006000656324
C	-2.32259289965241	2.04440511889797	-0.17395008024041
C	-3.75300099531995	2.08986137740576	-0.29120175010630
H	-4.33104056155534	2.99945143677047	-0.42657754458718
C	-4.21704310831787	0.81046062749512	-0.22377851600783

H	-5.24729291279064	0.47399751143393	-0.29455291395003
C	-3.08531805710824	-0.05932292016658	-0.06410664005196
C	-3.14051573953307	-1.45977283647593	0.00561924213816
C	-4.49537296879127	-2.08803870197562	-0.00935401236995
C	-4.93800266045821	-2.78847028584726	-1.13543034124963
H	-4.28900619225173	-2.86893949791390	-2.01005729398492
C	-6.20177816994834	-3.37106285115731	-1.14788098347181
H	-6.54074238663952	-3.91109239814975	-2.03391944666776
C	-7.03247994065529	-3.26195367117141	-0.03549256639614
H	-8.02304112423198	-3.72045154115148	-0.04567101965647
C	-6.59719946705962	-2.56658176904030	1.08980462410802
H	-7.24333646034799	-2.48198894121232	1.96557267734763
C	-5.33509767409007	-1.98044562227321	1.10319526718848
H	-4.99155485224019	-1.43972240418076	1.98762780433203
C	-2.09381730308422	4.49958797338686	-0.36146246849324
C	-2.00848023228524	5.20531511343148	-1.56532745564474
H	-1.48392824782005	4.76151952962549	-2.41413810452538
C	-2.59781909715624	6.46020441119688	-1.68620472350977
H	-2.53089563166642	7.00121885079855	-2.63194061751479
C	-3.27452058358709	7.02163719212269	-0.60622178923964
H	-3.73557157499088	8.00642382801103	-0.70183214726244
C	-3.36288841512137	6.32393057070291	0.59571759081117
H	-3.88945224087848	6.76156807549534	1.44590321914164
C	-2.77680469396354	5.06765048607883	0.71806238425357
H	-2.84199051403657	4.52242552012699	1.66211860515286
O	0.07150715988498	-0.01283244056268	1.96317941198335
C	-0.48051578499468	0.99870896189299	2.76531207495752
H	-0.03876846827980	1.99072669167680	2.56139016881803
H	-1.57645459502467	1.07817774934895	2.65330763082789
H	-0.26613920606037	0.74583586613232	3.81415540167500

References

1 P. J. Angiolillo and J. M. Vanderkooi, Electron paramagnetic resonance of the excited triplet state of metal-free and metal-substituted cytochrome c, *Biophys. J.*, 1995, **68**, 2505–2518.

2 Y. E. Kandrashkin, M. Di Valentin and A. van der Est, Reversible triplet energy hopping in photo-excited molecules: A two-site model for the spin polarization, *J. Chem. Phys.*, 2020, **153**, 094304.

3 K. M. Salikhov, R. Z. Sagdeev and A. L. Buchachenko, *Spin polarization and magnetic effects in radical reactions*, Elsevier, Netherlands, 1984.
